# USP10 drives cancer stemness and enables super-competitor signalling in colorectal cancer

**DOI:** 10.1038/s41388-024-03141-x

**Published:** 2024-10-23

**Authors:** Michaela Reissland, Oliver Hartmann, Saskia Tauch, Jeroen M. Bugter, Cristian Prieto-Garcia, Clemens Schulte, Sinah Loebbert, Daniel Solvie, Eliya Bitman-Lotan, Ashwin Narain, Anne-Claire Jacomin, Christina Schuelein-Voelk, Carmina T. Fuss, Nikolett Pahor, Carsten Ade, Viktoria Buck, Michael Potente, Vivian Li, Gerti Beliu, Armin Wiegering, Tom Grossmann, Martin Eilers, Elmar Wolf, Hans Maric, Mathias Rosenfeldt, Madelon M. Maurice, Ivan Dikic, Peter Gallant, Amir Orian, Markus E. Diefenbacher

**Affiliations:** 1 Protein Stability and Cancer Group, University of Wuerzburg, Department of Biochemistry and Molecular Biology, Wuerzburg, Germany; 2 Mildred-Scheel Early Career Cancer Center, Wuerzburg, Germany; 3 Division of Signal Transduction and Growth Control, DKFZ/ZMBH Alliance, German Cancer Research Center (DKFZ), Heidelberg, Germany; 4 Oncode Institute and Department of Cell Biology, Center for Molecular Medicine, University Medical Center Utrecht, Utrecht, Netherlands; 5 Molecular Signalling Group, Institute of Biochemistry II, Goethe University Frankfurt, Frankfurt am Main, Germany; 6 Rudolf-Virchow-Center for Integrative and Translational Imaging, University of Wuerzburg, Wuerzburg, Germany; 7 Department of Biochemistry and Molecular Biology, University of Wuerzburg, Wuerzburg, Germany; 8 Faculty of Medicine, TICC, Technion Haifa, Haifa, Israel; 9 Cancer Systems Biology Group, University of Wuerzburg, Am Hubland, Wuerzburg, Germany; 10 Department of Internal Medicine I, Division of Endocrinology and Diabetes, University Hospital, University of Wuerzburg, Wuerzburg, Germany; 11 Institute for Pathology, University of Würzburg, Wuerzburg, Germany; 12 Angiogenesis & Metabolism Laboratory, Berlin Institute of Health at Charité, Universitätsmedizin Berlin, Berlin Germany and Max Delbrück Center for Molecular Medicine in the Helmholtz Association, Berlin, Germany; 13 Stem Cell and Cancer Biology Laboratory, The Francis Crick Institute, London, UK; 14 Department of General, Visceral, Transplantation, Vascular and Paediatric Surgery, University Hospital, Julius-Maximilians-University of Wuerzburg, Wuerzburg, Germany; 15 Amsterdam Institute of Molecular and Life Sciences, Amsterdam, Netherlands; 16 Institute of Lung Health and Immunity, Helmholtz Center, Munich, Germany; 17 German Center for Lung Research, DZL, Germany; 18 Ludwig Maximilian University Munich, Munich, Germany; 19 Present Address: Max Planck Institute for Molecular Genetics, Berlin, Germany

**Keywords:** Cancer genetics, Ubiquitylation

## Abstract

The contribution of deubiquitylating enzymes (DUBs) to β-Catenin stabilization in intestinal stem cells and colorectal cancer (CRC) is poorly understood. Here, and by using an unbiassed screen, we discovered that the DUB USP10 stabilizes β-Catenin specifically in APC-truncated CRC in vitro and in vivo. Mechanistic studies, including in vitro binding together with computational modelling, revealed that USP10 binding to β-Catenin is mediated via the unstructured N-terminus of USP10 and is outcompeted by intact APC, favouring β-catenin degradation. However, in APC-truncated cancer cells USP10 binds to β-catenin, increasing its stability which is critical for maintaining an undifferentiated tumour identity. Elimination of USP10 reduces the expression of WNT and stem cell signatures and induces the expression of differentiation genes. Remarkably, silencing of USP10 in murine and patient-derived CRC organoids established that it is essential for NOTUM signalling and the APC super competitor-phenotype, reducing tumorigenic properties of APC-truncated CRC. These findings are clinically relevant as patient-derived organoids are highly dependent on USP10, and abundance of USP10 correlates with poorer prognosis of CRC patients. Our findings reveal, therefore, a role for USP10 in CRC cell identity, stemness, and tumorigenic growth by stabilising β-Catenin, leading to aberrant WNT signalling and degradation resistant tumours. Thus, USP10 emerges as a unique therapeutic target in *APC* truncated CRC.

## Introduction

Colorectal cancer (CRC) is the third most common cancer cases and 8–9% estimated deaths in 2021 [1]. It is widely accepted that environmental risk factors, such as diet, obesity, low level of exercise, alcohol and tobacco consumption increase the risk of developing CRC [2]^222^. Besides these environmental risk factors, inherited forms of CRC, characterized by distinct mutations, occur at a lower frequency [3]. Although most patients have an overall good survival after diagnosis, only 10% patients with progressed disease survive longer than 5 years [4]. This poor prognosis for late-stage patients punctuates the necessity of revealing novel and exploitable vulnerabilities in CRC for both sexes, with 8% estimated new cases.

In 80% of cases, CRC is characterized by hyperactivation of WNT signalling [5]. This is predominantly caused by truncating mutations in the tumour suppressor gene *Adenomatous Polyposis Coli* (*APC*). These truncating mutations are causative to the impaired ability of the WNT destruction complex to degrade the WNT effector β-Catenin, causing the onset of degradation resistant tumours, or short, DRTs [5, 6]. Several ‘hotspot’ mutations have been described for *APC*, including the catenin inhibitory domain (CID), resulting in various variants of APC truncation identified in patients [7]. This domain contains the β-Catenin binding 20 amino acid repeats (20 AAR). Consequently, *APC*-truncated tumour cells accumulate the co-activator β-Catenin, which translocates to the nucleus, displaces the TLE co-repressor and associates with the transcription factor TCF-4/LEF-1 to promote the expression of WNT pathway target genes [8, 9]. The accumulation of β-Catenin results in elevated cellular proliferation independent of WNT ligand availability and is believed to be a major event in disease onset of CRC [10, 11].

Truncating mutations within *APC* impact β-Catenin abundance or activity and subsequently oncogenic transformation [9, 12–15], as these cells only exhibit a limited ability to ubiquitylate β-Catenin [7]. Despite a basal post translational modification (PTM) activity retained by truncated APC [7], several E3 ligases were described in the past to modulate β-Catenin ubiquitylation independent of the WNT-destruction complex [16, 17], including, but not limited to, HUWE1 [18], FBXW7 [19], JADE-1 [20], UBR5 [21] and RNF4 [22].

Apart from ubiquitin ligases, several deubiquitylating enzymes (DUBs) were reported to deubiquitinate β-Catenin [23], such as USP7 [13] or USP20 [24], thereby extending the regulation of β-Catenin activity or abundance. However, whether the APC truncation status impacts the stabilization of β-Catenin by DUB enzymes is unknown. The possibility arises that truncated APC not only prevent the physiological degradation of β-Catenin, but also serves as a scaffold for β-Catenin regulating DUBs, enhancing β-Catenin accumulation in CRC, in an APC truncation specific manner. We investigated whether partial loss of discreet domains within APC allow de novo protein-protein interactions, towards which tumours could become addicted.

Here, we identified the DUB Ubiquitin Specific Protease 10 (USP10) as a direct binder of β-Catenin. The protein interaction between the DUB and the WNT effector only occurs in cells which have lost all AAR domains within *APC*, mutations present in around 30% of all CRC patients [25, 26]. USP10 is crucial in the tumorigenic signalling of β-Catenin and its abundance strongly enhanced WNT target gene expression and is required to maintain a *stemness-like* expression profile, in CRC and murine tumour organoids, respectively. Moreover, this mechanisms is evolutionarily conserved as elimination of USP10 suppressed the gut progenitor hyperproliferation phenotype and reduced survival in a homozygous *Apc* (*Apc*^*Q8/Q8*^) truncated *D.melanogaster* model [27]. Controlling USP10 abundance suppressed patient organoid growth and WNT and EMT signalling, while upregulating the unfolded stress response. Loss of USP10 reduced NOTUM signalling [28, 29], a prerequisite for competitive tumour cell growth, and prevented tumour engraftment and growth in vivo.

Overall, our study demonstrates that USP10 stabilises β-Catenin, promotes WNT-signalling and cancer stemness, essential to tumour onset and development in an *APC*^*ΔAAR*^-truncation dependent manner. Taken together our results suggest that targeting USP10 could thus be a viable treatment option for the large cohort of CRC patients carrying *APC*^*ΔAAR*^-truncating mutations.

## Results

### USP10 is a novel regulator of β-Catenin signalling in CRC and correlates with poor patient survival

In colorectal cancer, increased protein stability of the WNT effector β-Catenin is critical for tumorigenesis and mediated either by loss of function mutations and truncations within the *APC* gene or via mutations of the degron motive within *CTNNB1*, the gene encoding β-Catenin. While dysregulation or mutation of upstream canonical regulators of β-Catenin result in aberrant WNT target gene expression patterns due to increasing the protein levels β-Catenin [23, 30], did we wonder if the ubiquitylation of the WNT effector itself is altered [16], as several additional ubiquitin-regulatory pathways have been reported in the past that could contribute to oncogenesis in CRC.

**Fig. 1:**
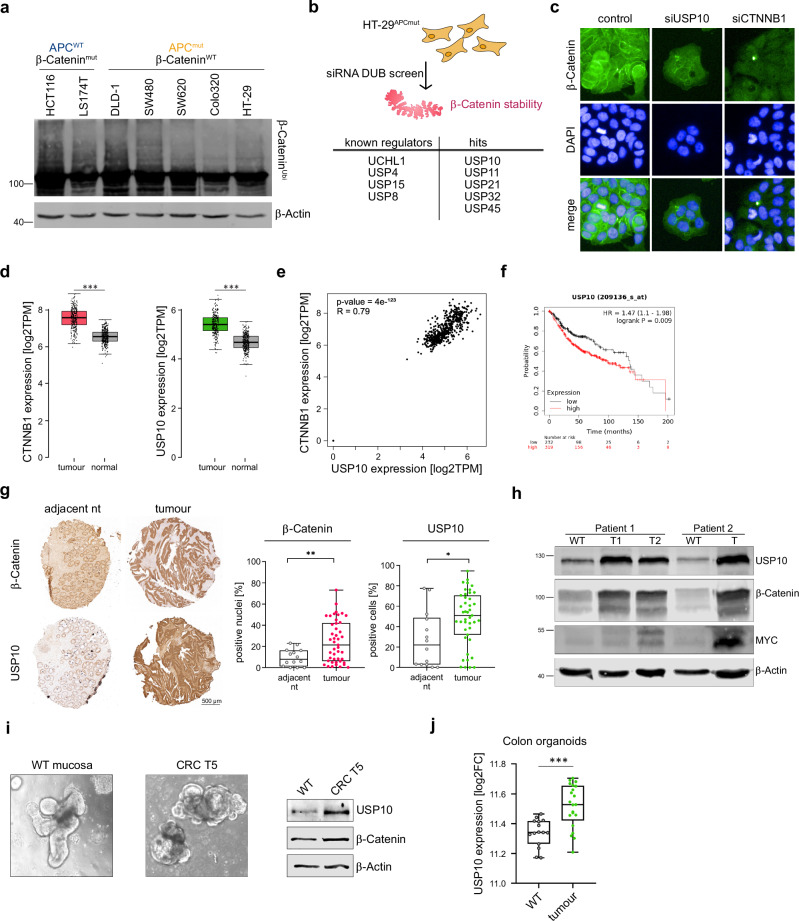
USP10 is a novel regulator of β-Catenin signalling in CRC and correlates with poor patient survival. **a** Tandem Ubiquitin Binding Entity (TUBE) assay of endogenous poly-ubiquitylated proteins, followed by immunoblotting against endogenous β-Catenin in human CRC cell lines with varying mutations. HCT116 and LS174T mutant for β-Catenin, DLD-1, SW480, SW620, Colo320 and HT-29 mutant for *APC*. β-Actin served as loading control. **b** Schematic model of siRNA DUB library screen conducted in *APC* mutant HT-29 cells. Cells were transfected with 4 individual siRNA against DUBs and 48 h post transfection immunofluorescence against endogenous β-Catenin was analysed via Operetta high-content microscope imaging. *n* = 3. DAPI served as nuclear marker. Identified known and new putative regulators of β-Catenin are highlighted. **c** Representative immunofluorescent images of endogenous β-Catenin (green) upon siRNA mediated knock-down of NTC (control), *CTNNB1* and *USP10*, respectively. DAPI served as nuclear marker (blue). **d** Expression of *CTNNB1* and *USP10* in non-transformed (normal) and CRC (tumour) samples. Publicly available data from GEPIA. COAD (*n* = 275) and GTEx (*n* = 349) data were displayed as boxplots for *USP10* and *CTNNB1* expression. *P*-values were calculated using one-way ANOVA. Data was visualised using the online tool www.gepia.cancer-pku.cn. ***p < 0.001. **e** Correlation of gene expression between *CTNNB1* and *USP10* in human CRC. R: Spearman’s correlation coefficient. *n*^T^ = 275, *n*^N^ = 349. Data was visualised using the online tool www.gepia.cancer-pku.cn. **f** Publicly available patient survival data of CRC patients are stratified by relative expression of *USP10*. *n* = 206 (low) and *n* = 26 (high). Survival correlation analysis was performed using R2: Genomics Analysis and Visualization Platform, using the Tumour Colon - Smith dataset. **g** Representative images of immunohistochemistry (IHC) staining of a Tissue Micro Array (TMA) from CRC patients, comprising adjacent non-transformed tissue (adjacent nt) and CRC samples against β-Catenin and USP10. *P*-values were calculated using Mann–Whitney U test. *p < 0.05; **p < 0.005. **h** Immunoblotting of endogenous abundance of USP10, β-Catenin and MYC in non-transformed (WT) and patient matched CRC tumour samples (T) from two individual patients. β-Actin served as loading control. **i** Representative brightfield images of patient derived intestinal organoids, comprising either wild type (WT mucosa) or tumour derived organoids (CRC T5), respectively. Immunoblotting of endogenous abundance of USP10 and β-Catenin of patient organoids. β-Actin served as loading control. **j** Expression of USP10 in non-transformed (WT) and CRC patient derived organoids (tumour). Analysis was performed using R2: Genomics Analysis and Visualization Platform, using the Organoid - Clevers dataset. *P*-values were calculated using Mann–Whitney U test. ***p < 0.001.

To investigate whether the nature of *CTNNB1* or APC mutations impact the stability and ubiquitylation of β-Catenin in CRC, did we conduct an endogenous ubiquitin TUBE (tandem ubiquitin binding entity) assay in a panel of human CRC lines, comprising β-Catenin mutant lines (HCT116 and LS174T), or cell lines varying in the truncation length of APC, DLD-1, SW480, SW620, Colo320 and HT-29, respectively (Fig. 1a). Remarkably, and irrespective of genetic alteration we were able to detect poly-ubiquitylation of β-Catenin (Fig. 1a). This suggested that protein stability of the WNT effector could be altered in a Ubiquitin Proteasome System (UPS) specific fashion likely by additional factors. To discover such regulators, we performed a human DUB siRNA screen in the *APC* truncated CRC line HT-29, followed by assessing the residual protein abundance of endogenous β-Catenin by immunofluorescence imaging using a high content screening microscope setup, relative to siRNA control transfected HT-29 (Fig. 1b, c). Analysis of the screen not only identified known regulators of β-Catenin stability, such as USP20 or UCHL1, but highlighted the deubiquitylase USP10 as a positive regulator of β-Catenin stability, along with previously identified DUBs (Fig. 1c and Supplementary Fig. S1a, b). Indeed, we verified that loss of USP10 resulted in the depletion of the cytosolic and nuclear pool of β-Catenin, as seen by immunofluorescence (Fig. 1c).

Since USP10 was not implicated in intestinal homeostasis and β-Catenin signalling, next, we determined expression level of USP10 by interrogating publicly available patient data of colorectal cancer [25]. While *USP10* was rarely mutated in CRC, it was predominantly upregulated, along with *CTNNB1*, when compared to adjacent wild-type tissue (Fig. 1d and Supplementary Fig. S1c), and *USP10* and *CTNNB1* demonstrated a significant degree of correlation of expression in CRC samples (Fig. 1e, *n*^T^ = 275, *n*^WT^ = 349, Spearman coefficient R = 0.79). Remarkably, elevated expression of *USP10* in CRC is a strong indicator of overall poor patient survival in CRC (Fig. 1f), especially in the molecular subtypes CMS2-4 (Fig. S1e). Prompted by this observation, we next studied USP10 and β-Catenin levels by Immunohistochemistry (IHC) of tissue micro arrays (TMA) of CRC patients comprising non-transformed and tumour tissue. Not only was a difference in tissue architecture observed in CRC tissue samples, but USP10 and β-Catenin indeed showed a significant upregulation in CRC when compared to the adjacent tissue (Fig. 1g). Elevated protein abundance was furthermore analysed by using human samples from CRC resection surgeries, subjected to immunoblotting against endogenous USP10, β-Catenin and MYC. Non-transformed, adjacent tissue served as control. USP10, β-Catenin and the oncogene MYC were increased in tumour-samples compared to matched non-transformed tissue samples (Fig. 1h).

Publicly available data did highlight that expression of *USP10* and *CTNNB1* were elevated irrespective of CRC stage (Supplementary Fig. S1f). This was further validated using single cell sequencing data from two individual datasets, which demonstrated a tumour-specific increase in USP10 expression when compared to non-transformed, normal tissue (Supplementary Fig. S1g) as well in spatial transcriptomic data from a publicly available dataset (https://www.10xgenomics.com/datasets/visium-hd-cytassist-gene-expression-libraries-of-human-crc, Supplementary Fig. S1 extended). We tested this observation in human and murine intestinal wild type and tumour organoid models regarding the regulation of USP10 (Fig. 1i). Similar to the observation in patent-derived primary resected CRC tumours, the endogenous protein levels of USP10 was significantly increased in tumour derived organoids, compared to non-oncogenic (Fig. 1i). This was further supported by analysing publicly available expression data of patient derived non-oncogenic and CRC organoids [31] (Fig. 1j).

These data propose that USP10 is a novel regulator of β-Catenin stability, and a putative involvement of USP10 in WNT signalling, intestinal homeostasis and carcinogenesis.

### Genetically engineered murine models of intestinal cancer demonstrate the upregulation of USP10 as an early event in CRC formation

**Fig. 2:**
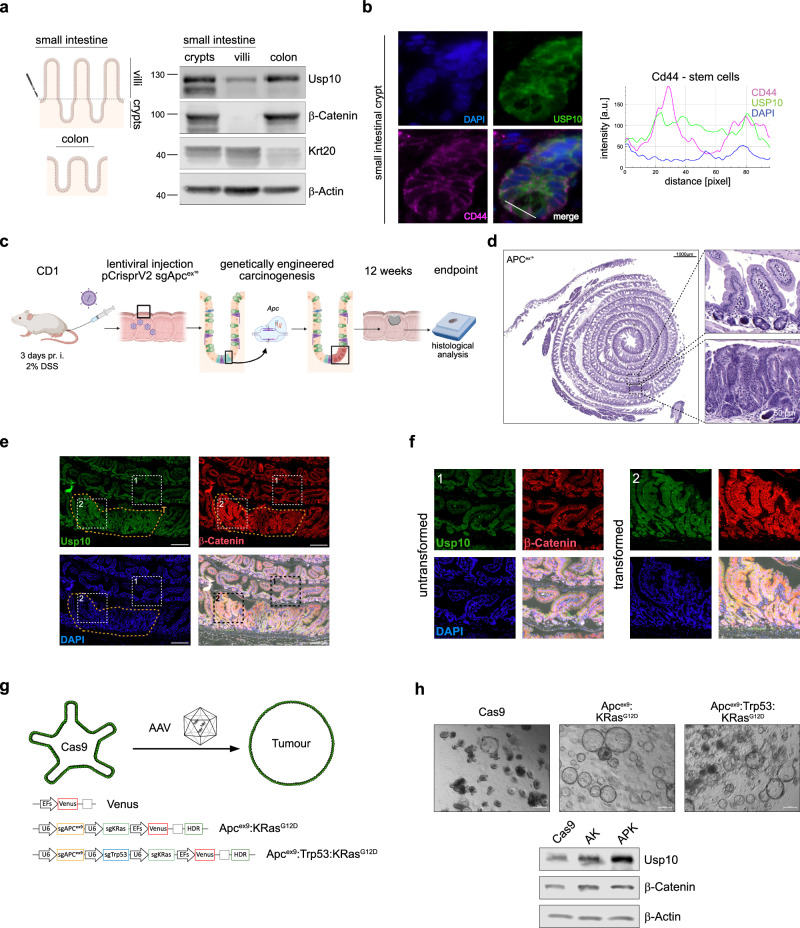
Genetically engineered murine models of intestinal cancer demonstrate the upregulation of USP10 as an early event in CRC formation. **a** Schematic representation of murine small intestine and colon. Villi were scratched from the intestine and small intestinal and colonic crypts were isolated using EDTA. Isolated tissue from two individual mice was analysed for endogenous abundance of USP10, β-Catenin and Krt20. β-Actin served as loading control. (*n* = 2). **b** Representative immunofluorescent images of WT intestinal crypts of endogenous USP10 (green) and crypt cell specific markers. Upper panel: Lysozyme (magenta) marks Paneth cells. Lower panel: Cd44 (magenta) labels stem cells. DAPI served as nuclear marker (blue). White line indicates stretch of fluorescence quantification. Histogram of fluorescence over indicated length. **c** Schematic representation of acute in vivo CRC onset in wild type CD1 animals using colorectal instillation of lentivirus particles encoding sgRNA against murine *Apc*, targeting exon 10 (*Apc*^*ex10*^), and constitutive expression of SpCas9. Viral backbone was pLenti-CRISPR-V2. pr.i. - pre infection. **d** Haematoxylin and eosin (H&E) staining of CRISPR mediated tumour onset in CD1 animals, 12 weeks post intracolonic instillation of virus. Insets highlight either non-transformed adjacent tissue or primary tumour upon *Apc* deletion. **e** Representative immunofluorescent images of mice shown in **a** and **b** of endogenous USP10 (green) and β-Catenin (red). DAPI served as nuclear marker (blue). Insets highlight either untransformed (1) or transformed (2) regions. Intensity of β-Catenin and USP10 staining was quantified using QuPath software. *P*-values were calculated using Mann–Whitney test. Individual cells/values are highlighted as dots. ***p < 0.001. **f** Insets from **c**. High magnification immunofluorescent images of intestines of CRISPR infected animals for USP10 (green) and β-Catenin (red). DAPI served as nuclear marker (blue). **g** Schematic representation of CRISPR-engineered murine CRC models using Adeno-associated viruses (AAV) to deliver sgRNA and HDR templates to either truncate endogenous *APC* within exon 9, *Trp53* or point mutate endogenous *Kras* to *Kras*^*G12D*^. **h** Representative brightfield images of murine intestinal organoids, comprising either wild type (Cas9), after targeting and growth factor depleted selection upon CRISPR engineering of *Apc* exon 9 (*Apc*^*ex9*^) and *KRas* to *KRas*^*G12D*^ *(AK)*, or upon co-deletion of *Trp53 (APK)*, respectively. Immunoblotting of endogenous abundance of USP10 and β-Catenin in Cas9, *AK* and *APK* organoids. β-Actin served as loading control.

To further investigate the expression of USP10 in the intestine, we examined weather USP10 is expressed in the intestinal stem cell niche and analysed unperturbed crypts (Fig. 2a, b and Supplementary Fig. S2a, b). Using fractionation of murine small intestine followed by immunoblotting we observed an enrichment of USP10 in crypts over villi (Fig. 2a). We confirmed that USP10 was abundant in intestinal crypts when compared to the villus (Fig. 2b and Supplementary Fig. S2a, b), and nuclear localised in intestinal stem cells using immunofluorescence (USP10^+^/β-Catenin^nuclear^; USP10^+^/Cd44^high^; USP10^+^/Lysozyme^-^; Fig. 2b and Supplementary Fig. S2b). This observation was further confirmed by analysing publicly available spatial transcriptomic data of murine intestine (https://www.10xgenomics.com/datasets/visium-hd-cytassist-gene-expression-libraries-of-mouse-intestine, Supplementary Fig. S2 extended).

Intrigued by the expression of USP10 in unperturbed intestinal tissue, we tested whether the abundance of USP10 is enriched upon transformation of intestinal cells. First, we performed histopathologic analysis of individual tumours in animals, where carcinogenesis was induced either by acute CRISPR editing of *Apc*, causing a truncation at exon 10 (*APC*^*ex10*^), or by loss of heterozygosity of *Apc* in a well-established mouse model of spontaneous intestinal cancer, *Apc*^*min/+*^ (Fig. 2c–f, Supplementary Fig. S2c–f). Irrespective of genetic alteration of *Apc*, causal to carcinogenesis we observed that the protein level of USP10 was significantly upregulated in tumours within the GI tract, along with elevated protein levels of β-Catenin, when compared to non-transformed or non-oncogenic intestinal epithelium, respectively (Fig. 2e, f and Supplementary Fig. S2e, f).

Next, we wondered if discreet genetic alterations could be a contributor to USP10 upregulation. To address this question, we used murine wild type organoids (*Cas9*) and employed CRISPR gene editing to generate *Apc:Kras*^*G12D*^ (*AK*) or *Apc:Trp53:Kras*^*G12D*^ (*APK*) organoids (Fig. 2g, h and Supplementary Fig. S2g). Loss of *Apc* induced morphologic changes of wild type murine organoids and alleviated the requirement for WNT activating components in the growth medium. This is in accordance with previous reports, and likewise mutation of *Kras* to *Kras*^*G12D*^ upregulated endogenous Erk1/2 signalling and alleviated the requirement for EGF supplementation (Supplementary Fig. S2g) [32]. In accordance the protein level of USP10 and β-Catenin was significantly enriched in transformed organoids when compared to parental control (Fig. 2h).

Thus, the upregulation of USP10 in colorectal cancer is an early event, caused by oncogenic transformation irrespective of genetic driver complexity, and coincides with elevated abundance of the WNT effector β-Catenin.

### Truncation of APC allows for de novo protein-protein interaction between USP10 and β-Catenin in CRC

**Fig. 3:**
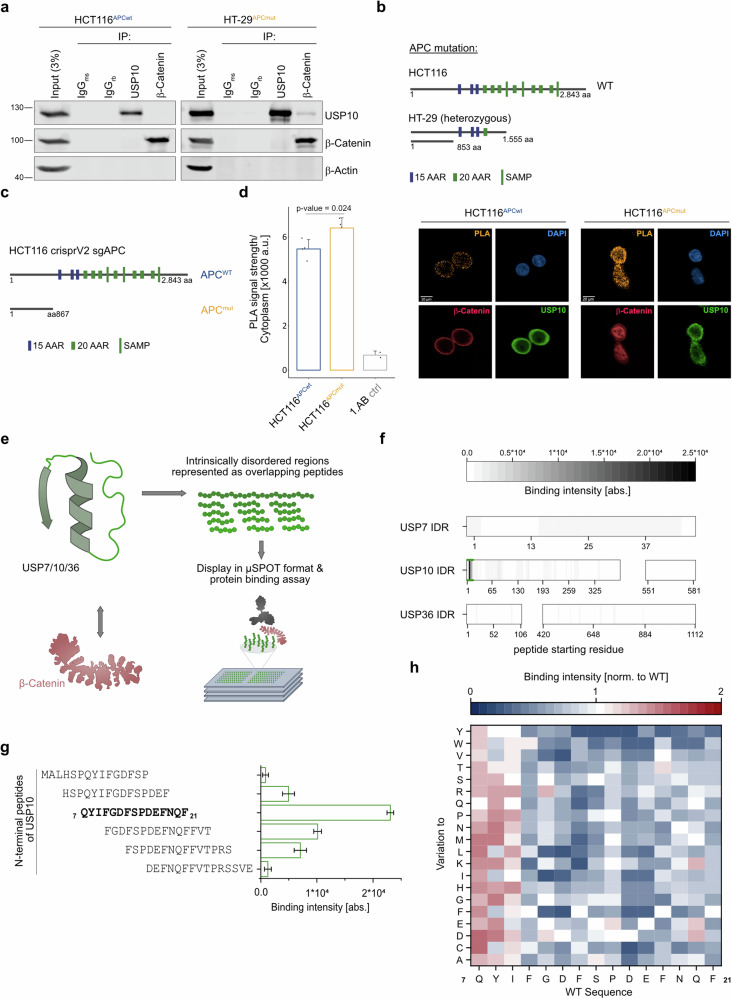
Truncation of APC allows for de novo protein-protein interaction between USP10 and β-Catenin in CRC. **a** Representative input and endogenous co-immunoprecipitation of USP10 and β-Catenin in human CRC cell lines either wild type for APC, HCT116^APCwt^, or truncated HT-29^APCmut^. IgG served as antibody specificity control. β -Actin served as loading control. Input represents 3% of total loading. *n* = 3. **b** Schematic representation of truncating mutations reported in the *APC* gene in the CRC cell lines HCT116 and HT-29. Dark blue box = 15 AAR domains, green small boxes = 20 AAR domains, large green boxes = SAMP domains. 15- and 20-AAR = β-Catenin amino acid repeats; SAMP = Axin binding sites. Images adapted from the publicly available database www.uniprot.org. **c** Schematic model of acute truncation of *APC* at amino acid 867 in HCT116 via CRISPR gene editing. **d** Bargraph of proximity ligation assay (PLA) between USP10 and β-Catenin in either *APC wild type (APC*^*wt*^*)* or *APC*^*867*^ truncated (APC^mut^) HCT116. Data analysed from more than 750 cells over two independent experiments per condition. *P*-values were calculated using Mann–Whitney U test. Representative immunofluorescent images of endogenous USP10 (green), β-Catenin (red) and the corresponding PLA (mustard) in either *APC*^*wt*^ of *APC*^*mut*^ HCT116. DAPI served as nuclear marker. *n* = 2. **e** Schematic overview of the in vitro binding assay in µSPOT format. Intrinsically disordered regions of USP7, USP10 and USP36, respectively, were determined by <50 pLDDT score in their individual AlphaFold2 structural prediction and represented as 15 mer peptides overlapping 12 or 11 amino acids. For binding assays, µSPOT slides bearing the peptide library were incubated with recombinantly expressed and purified β-Catenin. β-Catenin binding to the on-chip peptides was detected by immunostaining with a chemiluminescent readout. **f** Overview of binding intensities of recombinant β-Catenin towards discreet unstructured regions of USP7, USP10 and USP36. Gaps indicate the presence of structured domains within the DUBs. Colour code indicates binding intensity. Peptides with the globally highest binding intensity (N-terminal region of USP10 residues 7–21) are underlined in green and represented as a bar graph in panel **g**). Mean of *n* = 3. **g** Identified amino acid sequence within the N-terminal unstructured part of USP10 binding to recombinant β-Catenin. Bar graph shows binding intensity (abs. = absolute intensity). Mean of *n* = 3 with corresponding standard deviation. **h** Full positional scan of the most prominent β-Catenin binding hotspot USP10^7-21^ identified in the overlapping scan (panel **g**). Each residue of the peptide sequence was systematically varied to every other proteogenic amino acid and their β-Catenin binding intensities are shown relative to the wildtype sequence. Note that amino acid variations for certain positions result in drastic reductions in binding intensity compared to the wildtype sequence, thus suggesting direct interactions of the respective sidechains with β-Catenin. Mean of *n* = 3.

We hypothesized that β-Catenin directly interacts with USP10 in CRC and tested whether mutations within either β-Catenin or *APC* are a prerequisite to enable a protein-protein interaction. To this end, we co-immunoprecipitated endogenous USP10 and β-Catenin in the human CRC lines HCT116 (*CTNNB1*^*mutant*^*/APC*^*wildtype*^) and HT-29 (*CTNNB1*^*wildtype*^*/APC*^*truncated*^) by either immunoprecipitating USP10 or β-Catenin first, followed by probing against the putative novel interaction partner (Fig. 3a, b). While USP10 and β-Catenin were singly immunoprecipitated in HCT116, no co-precipitation was observed. In contrast, USP10 co-immunoprecipitated with endogenous β-Catenin in HT-29 cells (Fig. 3a). This observation highlighted the possibility that the truncation status and length of APC is involved in the interaction between USP10 and β-Catenin. To examine this point, we used a panel of human CRC lines, comprising LS174T, DLD-1, SW480, SW620, Caco-2 and Colo320, which harbour varying truncation mutations within *APC* (Supplementary Fig. S3b, c). Endogenous co-immunoprecipitation of USP10 and β-Catenin only occurred in Colo320, confirming that a proximal truncation in *APC* is a prerequisite, as cell lines carrying distal deletions, such as DLD-1, Caco-2 and SW480/SW620, failed to co-immunoprecipitate USP10 with β-Catenin (Supplementary Fig. S3b, c). The truncation status within APC could have potential therapeutic implications, since analysing patient survival data and stratification of CRC patients regarding APC status (truncation within first 1000 amino acids or after, or carrying point mutations), indicated a trend towards shorter survival for short APC variant carriers (Supplementary Fig. S3a).

Furthermore, based on the genetic alterations reported for HT-29 and Colo320, we concluded that the 15- and 20 β-Catenin binding amino acid Armadillo repeats (AAR) domains within APC are required to directly compete for binding of USP10 to β-Catenin. Hence, we postulate that the putative de novo interaction between USP10 and β-Catenin requires the loss of the AAR domains. Proximity ligation assays (PLA) between USP10 and β-Catenin in either control or CRISPR mediated APC-truncated HCT116 cells confirmed that *APC* competed with USP10 for binding to β-Catenin (Fig. 3c, d).

To further interrogate the interaction and map the USP10 binding site required for β-Catenin interaction, we conducted a µSPOT protein binding assay [33]. Here, the intrinsically disordered regions (IDR) of USP10, along with the IDR sequences of a known β-Catenin binder, USP7 [13], or USP36 (an additional DUB comprised of large unstructured regions), were displayed as overlapping peptide libraries and probed with recombinant β-Catenin (Fig. 3e). We identified USP10 residues ^7^QYIFGDFSPDEFNQF^21^ (Fig. 3f–h) to mediate direct and robust binding to β-Catenin. Intriguingly, by assessing the binding affinity of β-Catenin towards its known interactors Axin1, APC or TCF4 we found that the presence of the USP10 peptide did interfere with binding of β-Catenin to AXIN1 and APC, pointing towards a high affinity of β-Catenin towards USP10 and TCF4 (Supplementary Fig. S3d, e). Co-immunoprecipitation of endogenous β-Catenin in HT-29 transiently transduced with either control, wild type USP10 or USP10 depleted for amino acids 7–21 (USP10^Δ7-21^) revealed that the point mutant had a reduced interaction potential with endogenous β-Catenin (Supplementary Fig. S3f). This was further studied using AlphaFold2 MultimerV1.0 (AF2M) [34], with the complete sequences of USP10 and β-Catenin as input, predicts the same residues within USP10 to engage with β-Catenin, as identified by µSPOT protein binding assay (Supplementary Fig. S3g, h). Remarkably, this binding site is overlapping with APC and AXIN1 binding to β-Catenin (Supplementary Fig. S3f, g).

Taken together, we discovered a direct USP10-β-Catenin interaction as well as that both USP10 with APC compete for the same β-Catenin binding site. Thus, lending a molecular explanation for the observed indirect β-Catenin stabilizing effect of APC truncations.

### Acute deletion of USP10 in intestinal stem cells of D. melanogaster rescues hyperplasia and lethality of the Apc^Q8/Q8^ model

**Fig. 4:**
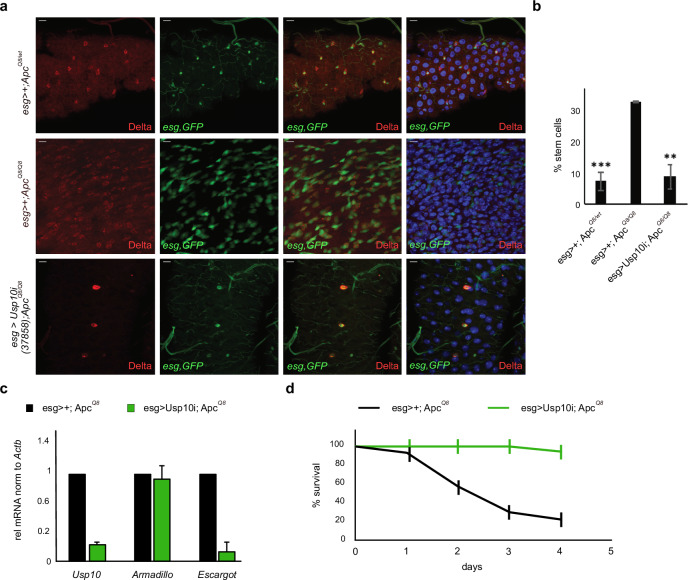
Acute deletion of USP10 in intestinal stem cells of D.melanogaster rescues hyperplasia and lethality of the Apc^Q8/Q8^ mutant flies. **a** Representative immunofluorescence of fly midguts. *Apc*^*Q8/+*^ heterozygotes are highly similar to wildtype midguts (not shown). Midguts of homozygous *Apc*^*Q8*^ mutants exhibit hyperproliferation of ISC (positive for the intestinal stem cell marker Delta (red)). Elimination of USP10 using USP10 inverted repeats (UAS-IR) suppresses the progenitor hyperproliferation phenotype observed in midguts of homozygous *Apc*^*Q8*^ mutants. **b** Quantification of total stem cell abundance in all three conditions. Significance as compared to “esg > +; ApcQ8” was calculated using one-way ANOVA. **p < 0.005; ***p < 0.001. **c** qRT-PCR analysis of the expression of *USP10*, *armadillo* and *escargot* in midguts isolated from either *Apc*^*Q8*^ or *Apc*^*Q8*^ *USP10*^*KD*^ flies. mRNA was normalised to *Actb*. Error bars represent standard deviation of 3 biological replicates. **d** Kaplan–Meier plot of adult survival of the indicated genotypes. *Apc*^*Q8*^ *n* = 24, *Apc*^*Q8:esg-USP10i*^ *n* = 17.

As USP10 and the entire Wnt pathway is highly conserved between species we used *D. melanogaster* to investigate its involvement in intestinal homeostasis and hyperproliferation upon loss of function mutations within *APC*, [35] (Similarity in aa: 379/821 - (46%), Identity in aa: 254/821 - (30%); Gaps: in aa:185/821 - (22%) https://www.flyrnai.org/cgi-bin/DRSC_prot_align.pl?geneid1 = 38103&geneid2 = 9100; Fig. 4 and Supplementary Fig. S4). Firstly, we assessed the impact of shRNA-mediated elimination of dUSP10 on intestinal progenitor homeostasis. Intestinal progenitor cells were marked by GFP expression, driven under the control of the *escargot* regulatory region (*esg*::GAL > GFP), and immunofluorescence against *armadillo*, the fly ortholog to β-Catenin, that is expressed in intestinal stem cells (ISCs, Supplementary Fig. S4a). Expression of shRNA against USP10 resulted in a marked reduction of ISCs when compared to a LacZ control shRNA (Supplementary Fig. S4a, b).

Moreover, we tested for a genetic interaction between *APC* truncation and USP10 in a tumour-like setting using the *Apc*^*Q8*^ hyperplasia model [36]. The allele *Apc*^*Q8*^ harbours a premature stop codon leading to a significant truncation of *Apc* and loss of the β-catenin binding sites. Immunofluorescent analysis of *D. melanogaster* midguts revealed that heterozygous loss of *Apc* had a minor effect on overall tissue homeostasis highly similar to wildtype midguts. In contrast, midguts derived from adult animals carrying a homozygous LOF truncating mutation within *Apc* (*Apc*^*Q8/Q8*^) presented an entirely disorganised intestine (Fig. 4a). This midgut was robustly populated by escargot-positive progenitors, many of them expressing the stem cell marker and Notch ligand Delta. (Fig. 4a, b). Transcriptional analysis of midguts isolated from *Apc*^*Q8/Q8 revealed*^ a significant increase in dUSP10 mRNA levels (Supplementary Fig. S4e). This is in alignment with the identified expression pattern observed in human and murine CRC and stresses a high degree of mechanistic similarities between the chosen model organisms. Knockdown of dUSP10, however, suppressed the stem cell and progenitor expansion observed in homozygous *Apc*^*Q8*^, and animals presented a midgut resembling a normal appearance (Fig. 4a, b). Analysis of isolated midguts from either *Apc*^*Q8/+*^, *Apc*^*Q8/Q8*^ *or Apc*^*Q8/Q8*^ flies expressing an shRNA against dUSP10 (*USP10i;Apc*^*Q8/Q8*^) in intestinal stem cells indicated a significant reduction in overall USP10 transcript abundance, along with reduced expression of the stem cell marker escargot (Fig. 4c and Supplementary Fig. S4e).

Lastly, we investigated the impact of dUSP10 deletion on overall survival in the background of *Apc*-truncation driven hyperplasia model. While the survival of heterozygous *Apc*^*Q8/wt*^ flies was similar to wildtype files, homozygous *Apc*^*Q8/Q8*^ mutation were characterized with a temperature-sensitive lethality (Fig. 4d and Supplementary Fig. S4f). Strikingly, expression of an shRNA against d*USP10* in intestinal progenitors (*USP10i;Apc*^*Q8/Q8*^*)* restored longevity, likely by negating the adverse effects on overall tissue homeostasis and growth imprinted by *Apc*^*Q8/Q8*^ (Fig. 4d).

These data demonstrate an epistatic genetic linkage between USP10 and truncated APC that is required for ectopic stem cell proliferation.

### USP10, via controlling β-Catenin protein stability, regulates WNT signalling and stemness signature genes

**Fig. 5:**
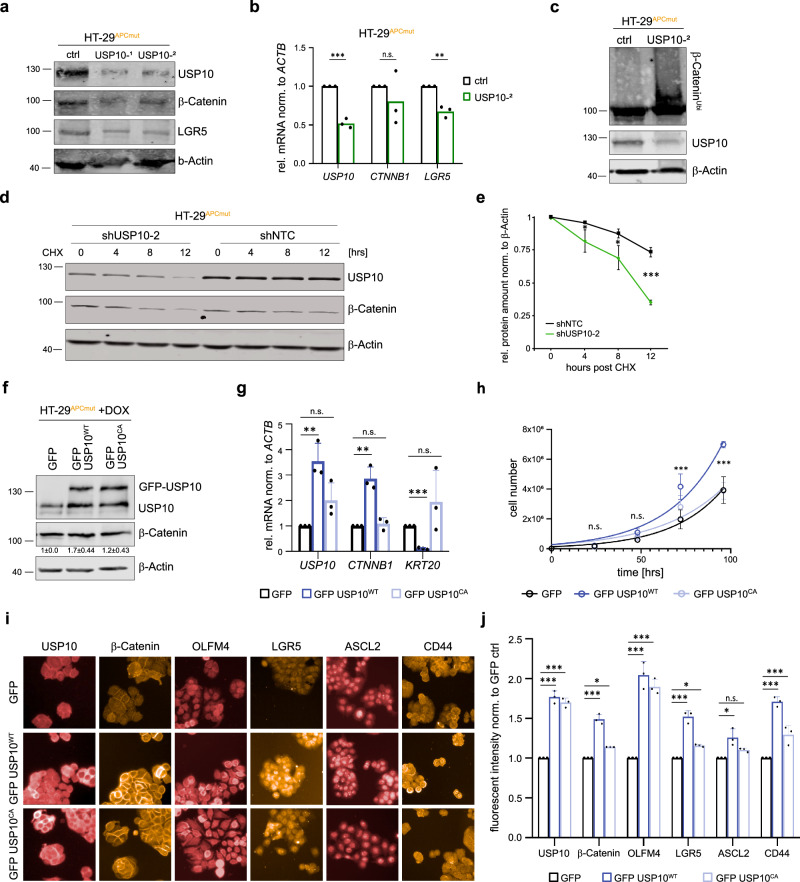
USP10 regulates WNT signalling and stemness signature genes via controlling β-Catenin protein stability. **a** Immunoblot against endogenous USP10, β-Catenin and LGR5 in *APC* mutant HT-29 cells upon CRISPR mediated depletion of USP10. Two different cell pools (USP10^−1^ and USP10^−2^) along with non-targeting control (ctrl) cells are shown. β-Actin served as loading control. *n* = 3. **b** Quantitative RT-PCR of *USP10*, *CTNNB1* and *LGR5* expression of HT-29 USP10 CRISPR pool (USP10^−2^) compared to control (ctrl) cells. Error bars represent standard deviation of *n* = 3 independent experiments. Significance was calculated using Student’s *t* test. **p < 0.005; ***p < 0.001. n.s. non-significant. **c** Tandem Ubiquitin Binding Entity (TUBE) assay of endogenous poly-ubiquitylated proteins, followed by immunoblotting against endogenous β-Catenin in HT-29 USP10 CRISPR cells (USP10^−2^). Immunoblot against endogenous USP10 is shown. β-Actin served as loading control. *n* = 2. **d** Cycloheximide (CHX) chase assay (100 μg/ml) of control (shNTC) or shUSP10-2 expressing HT-29 cells for indicated time points. Representative immunoblot analysis of USP10 and β-Catenin. β-Actin served as loading control. *n* = 3. **e** Quantification of relative protein abundance of β-Catenin, normalised to β-Actin, as shown in **d**. Significance was calculated using Student’s *t* test. *n* = 3 *p < 0.05; ***p < 0.001. **f** Representative immunoblot against endogenous USP10 and β-Catenin in *APC* mutant HT-29 cells upon DOX-inducible overexpression of GFP control (GFP), catalytical active GFP-USP10 (GFP USP10^WT^) and a catalytical inactive mutant of USP10 (GFP USP10^CA^). β-Actin served as loading control. (*n* = 3). **g** Quantitative RT-PCR of *USP10*, *CTNNB1* and *KRT20* expression of HT-29 cells overexpressing exogenous USP10. Error bars represent standard deviation of *n* = 3 independent experiments. Significance was calculated using Student’s *t* test. **p < 0.005; ***p < 0.001. n.s. non-significant. **h** Growth-curve of GFP USP10^WT^ and GFP USP10^CA^ overexpressing HT-29 cells compared to GFP control cells. Error bars represent standard deviation of *n* = 3 independent experiments. Significance was calculated using one-way ANOVA. ***p < 0.001. n.s. non-significant. **i** Representative immunofluorescence images of conditional USP10^WT^ and USP10^CA^ overexpression and GFP control in HT-29 cells. **J** Quantification of **i**. Mean intensity over well was measured and normalised to GFP control. Error bars represent standard deviation of *n* = 3. Significance was calculated using unpaired t-test. *p < 0.05; ***p < 0.001; n.s. non-significant.

To further elucidate the function of USP10 in CRC in the context of APC truncation, we deleted endogenous USP10 by co-targeting of exon 2 and 10 in HT-29 and HCT116, respectively (Fig. 5a and Supplementary Fig. S5a, b). Depletion of USP10 in HT-29 resulted in a marked reduction of β-Catenin, along with reduction in the CRC protein marker and WNT target gene LGR5 (Fig. 5a, b). Loss of USP10 enhanced overall ubiquitylation of β-Catenin (Fig. 5c) and accelerated protein turnover in HT-29 cells (Fig. 5d and quantified in 5e). Depletion of USP10 in HCT116 a cell line that harbour non-truncated *Apc*, however, had no effect on overall β-Catenin abundance nor ubiquitylation (Supplementary Fig. S5b–d), confirming the dependency of the USP10-β-Catenin interaction on *APC*-truncation. Interestingly, while cells deleted for USP10 by CRISPR mediated targeting did show reduced abundance and increased ubiquitylation of β-Catenin, longitudinal propagation of HT-29^ΔUSP10^ was not possible. Targeted cells within a heterogeneous cell pool were rapidly outcompeted by wildtype cells (Supplementary Fig. S5e). This is in line with previous reports of cell lethality upon USP10 loss [37]. To by-pass this long-term lethality, we used an inducible knock down system, comprising two independent shRNA against *USP10*, to acutely deplete the DUB in HT-29 (Supplementary Fig. S5f). USP10 depleted HT-29 showed a significantly reduced proliferation, when compared to control vector transduced cells (Supplementary Fig. S5g).

A stem cell niche-specific contribution of USP10 was further supported by analysing the whole proteome of HT-29 cells treated with either non-targeting (ctrl) or USP10 siRNA for 24 h (Supplementary Fig. S5h, i). Among the downregulated proteins were proteins associated with the stem cell niche, including TCF4 (TCF7L2), TNFRSF21, NOTCH2, LGR4, CD44, along with reduced protein level of the proto-oncogene MYC, a direct target of WNT signalling (Supplementary Fig. S5h).

To investigate the extent of regulation of the WNT effector β-Catenin by USP10, and using a gain-of-function approach, we conditionally overexpressed either wild type (USP10^WT^) or a catalytic inactive variant of USP10 (USP10^CA^) in the CRC line HT-29. Conditional increase in USP10 led to an increase in β-Catenin abundance on protein as well as mRNA level (Fig. 5f, g). The catalytic activity of USP10 is required to facilitate these effects on β-Catenin, as USP10^CA^ failed to stabilise β-Catenin (Fig. 5f, g). Expression of USP10 significantly enhanced overall proliferation of HT-29 cells, when compared to vector or catalytic inactive mutant control cells (Fig. 5h). Given that β-Catenin directly controls intestinal homeostasis and stem- and cancer cell maintenance, next, we tested if USP10 affects the expression of essential stem- and CRC pathways. Immunofluorescence imaging of HT-29 expressing either USP10^WT^ or USP10^CA^ demonstrated that proteins associated with the CSC stem niche, such as β-Catenin, OLFM4, LGR5, ASCL2 or CD44 were significantly upregulated in a USP10^WT^ dependent fashion (Fig. 5i, j).

These observations establish that USP10 regulates the ubiquitylation and abundance of β-Catenin in an *APC* truncation dependent manner, promoting the expression of WNT pathway and (cancer) stem cell signatures and CRC growth.

### USP10 is required to maintain CRC cell identity, stemness and tumour growth

**Fig. 6:**
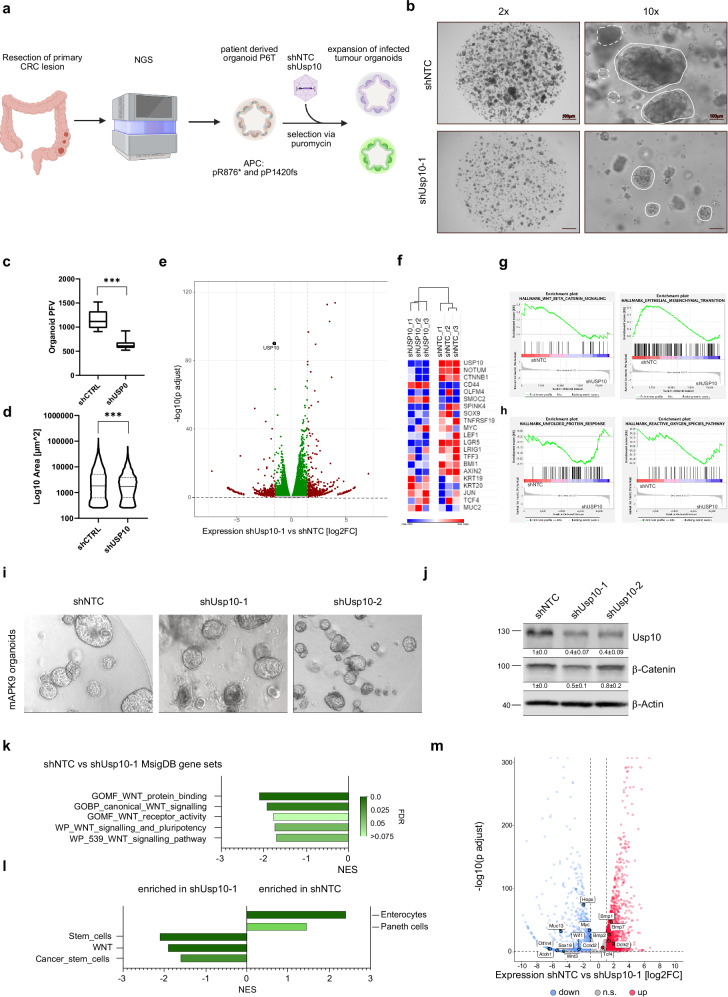
USP10 is required to maintain CRC cell identity, stemness and tumour growth. **a** Schematic overview of workflow for isolation, characterisation and silencing of USP10 in patient derived CRC organoid P6T (Oncode Organoid bank). **b** Representative brightfield images of stable transformed human P6T organoids infected with either shRNA against USP10 or with a non-targeting control. *n* = 10 field of view. Highlighted are individual and intact organoids. **c** Quantification of relative organoid number (per field of view) one week post infection with either a control (shNTC) or shUSP10. Statistical analysis was performed using unpaired t test. *p* < 0.0001. Images were quantified using QuPath (version0.4.2) and ImageJ (FIJI). Boxplots were generated using Graphpad Prism8. In box plots, the centre line reflects the median and the upper and lower box limits indicate the first and third quartiles. Whiskers extend 1.5× the IQR. *P*-values were calculated using Mann–Whitney U test. ***p < 0.0001. **d** Quantification of relative organoid size (per field of view) one week post infection with either a control (shNTC) or shUSP10. Statistical analysis was performed using unpaired t test. *p* < 0.0001. Images were quantified using QuPath (version0.4.2) and ImageJ (FIJI). Violinplots were generated using Graphpad Prism8. P-values were calculated using Mann–Whitney U test. ***p < 0.0001. **e** Volcano-plot of differential expressed genes upon knock-down of USP10 in human P6T organoids, relative to expression in shNTC infected control organoids. Significantly regulated genes are highlighted in red, respectively. USP10 is highlighted. *n* = 3. **f** Heatmaps showing expression of genes linked to WNT signalling, differentiation and NOTUM signalling in either shNTC or shUSP10 P6T organoids. *n* = 3. **g**, **h** GSEA analysis of P6T organoids expressing an shRNA sequence targeting USP10 or non-targeting control (shNTC). Changes in gene expression were analysed and enrichment plots for gene sets mapping to WNT signalling, EMT, UPR and ROS are shown. **i** Representative brightfield images of stable transformed murine *A*^*ex9*^*PK*^*G12D*^ organoids (APK9). Two different shRNAs against USP10 and shNTC expressing organoids were generated. **j** Representative immunoblot of USP10 and β-Catenin protein upon shRNA mediated knock-down of endogenous USP10. β-Actin served as loading control. Quantification was calculated from *n* = 3. **k** Gene set enrichment analysis of MsigDB gene sets, deregulated in shUSP10-1 compared to shNTC APK9 organoids. **l** Gene set enrichment analysis of intestinal specific gene sets, deregulated in shUSP10-1 compared to shNTC APK9 organoids. **m** Volcano-plot of differential expressed genes upon knock-down of USP10 in APK9 organoids. Up- and down-regulated genes are highlighted in red and blue, respectively. Genes-of-interest are labelled.

To investigate the clinical relevance and dependency of human CRC tumours towards USP10 in a patient-relevant setting, we used patient-derived organoids (Fig. 6a). The patient organoid line P6T carries mutations comparable to HT-29; a truncating mutation resulting in a short APC variant (R^876*^) and a longer variant (P^1420fs^), making it a suitable candidate to test USP10 dependency. 3 weeks post infection and selection with either a non-targeting control shRNA (shNTC) or an shRNA targeting USP10, patient-derived organoids were analysed (Fig. 6b–h). Loss of USP10 significantly reduced overall organoid numbers and size (Fig. 6c, d). Transcriptomic analysis of P6T^shNTC^ and P6T^shUSP10^ organoids revealed that USP10 is involved in the regulation of WNT signalling, differentiation and stem cell maintenance (Fig. 6f, g). Stem cell-related genes, such as LGR5, LEF1, AXIN2 or LRIG1 were reduced upon loss of USP10, while the expression of differentiation associated genes, such as MUC2 or KRT20, were enriched (Fig. 6f). Furthermore, loss of USP10 led to enriched gene sets associated with stress signalling, such as unfolded protein response and reactive oxygen species signalling in P6T tumour organoids (Fig. 6h). These observations are in line with the results obtained from HT-29 cells and clearly demonstrate that USP10 is involved in the maintenance/propagation of a pro-tumorigenic signature, supporting stem cell-like features of cells expressing high levels of USP10 that is required for the tumorigenic state.

In addition, we tested whether these observations are conserved in murine models of CRC using murine intestinal organoid cultures (Fig. 6m). *APK9* organoids were transduced with AAV encoding either shRNA against USP10 or non-targeting control, respectively (Fig. 6i–m and Supplementary Fig. S6a). Depletion of USP10 was confirmed by immunoblotting (Fig. 6j). Transcriptomic analysis of *APK9* organoids revealed that USP10 is required to maintain WNT signalling, as loss of USP10 significantly reduced this pathway (Fig. 6k–m). Knock down of USP10 resulted in reduced abundance of β-Catenin as well WNT signalling target genes, such as Myc and Ccnd2 (Fig. 6m and Supplementary Fig. S7b). Overall, reduction of USP10 in *APK9* organoids reduced signatures associated with stemness and induced the expression of differentiation gene signatures (Fig. 6k, l and Supplementary Fig. S6b).

These results show that USP10, in CRC at least, contributes to the control of differentiation and can be linked to intestinal cancer cell identity. Hence, USP10, via β-Catenin, promotes intestinal cancer stemness and propagation.

### Loss of USP10 opposes competitor signalling and restores a wild-typic niche

**Fig. 7:**
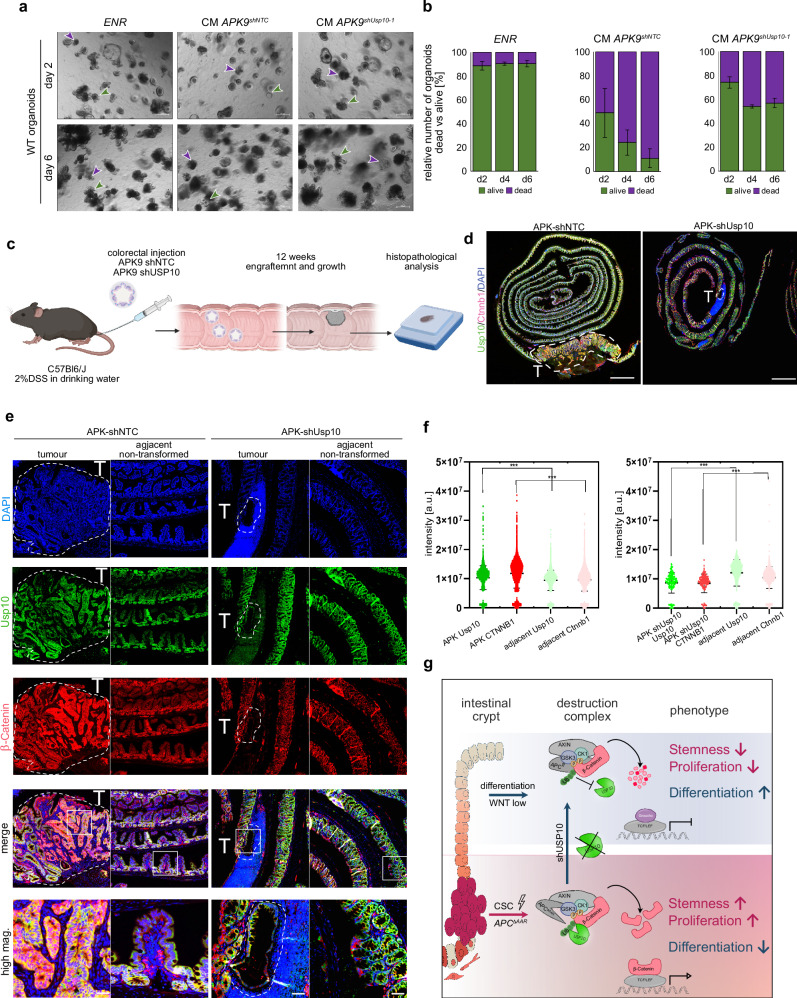
Loss of USP10 opposes competitor signalling and restores a wild-typic niche. **a** Representative brightfield images of wild-type (WT) organoids cultured in ENR medium, *APK*^*shNTC*^ *and APK*^*shUSP10-1*^ conditioned medium (CM), supplemented with EGF and R-spondin, for up to 6 days. Purple arrows indicate dead organoids, green arrows indicate living organoids. E – EGF, N – Noggin, R - R-spondin. *n* = 3. **b** Dead and alive organoids were counted and bar graphs represent percentage of alive vs dead organoids. Error bars represent standard deviation calculated from *n* = 3 independent experiments.Schematic representation of an in vivo organoid transplant model of APK shNTC or shUSP10, respectively. Adapted from refs. [39, 41, 62]. **c** Schematic representation of the in vivo organoid transplant model in immune-competent C57Bl6/J mice. **d** Representative merged immunofluorescent images of endogenous USP10 (green) and β-Catenin (red) of murine intestines upon organoid transplant. Encircled are tumours arising from engrafted somatic engineered APK organoids, either expressing an shNTC or shUSP10, respectively. DAPI served as nuclear marker. Scale bar represents 1 mm. **e** Individual higher magnification images of discreet tumours upon transplant as seen in d. USP10 (green) and β-Catenin (red) are shown. Highlighted are either tumour areas or adjacent, non-transformed tissue regions. Tumour area is encircled. Scale bar 200 μm. Zoom in image of merged image. Scale bar 50 μm. **f** Quantification of relative fluorescence intensity (arbitrary units a.u.) of endogenous USP10 and β-Catenin, either in shNTC or shUSP10 animals. Statistical analysis was performed using unpaired t test. ***p < 0.0001. Images were quantified using QuPath (version0.4.2). Boxplots were generated using Graphpad Prism8 and individual datapoints are shown. 1. **g** Schematic model. In APC-truncation driven CRC, loss of all AAR-domains in APC, enables a de-novo interaction of USP10 and β-Catenin. By stabilising β-Catenin, this interaction activates the transcription of β-Catenin target genes, thereby promoting a stem-like phenotype and proliferation and decreasing differentiation. Interfering with *USP10* expression by shRNA and/or CRISPR/Cas9 reverts this phenotype and leads to decreased stemness and proliferation and promotes differentiation.

Recently, it was shown that cancer cells eliminate the non-transformed intestinal stem cells by clonal competition derived by *Apc*-dependent *Notum* signalling, that induces the death of the naïve stem cells termed super competitor phenotype. This impact of the cancer cell on non-transformed neighbouring naive intestinal stem cells was shown to be crucial for tumour development [28, 29, 38]. Given the strong impact on WNT signalling and the extended control of β-Catenin by USP10, we examined whether USP10 is required for the super-competitor phenotype and, specifically, if silencing of USP10 could oppose this signalling axis. Towards this end, we cultured wild type organoids in the presence of pre-conditioned medium from either *APK*^*shNTC*^ or *APK*^*shUSP10*^ organoids and assessed wild type organoid survival (Fig. 7a, b and Supplementary Fig. S7a). While established wild type organoids grew in ENR medium, exposure to *APK*^*shNTC*^ derived medium rapidly resulted in wild type organoid loss (Fig. 7a, b and Supplementary Fig. S7a). Remarkably, when cultured in medium from *APK*^*shUSP10*^ organoids, most wild type organoids survived an extended time under these conditions (Fig. 7a, b and Supplementary Fig. S7a). Analysis of the transcriptome of *APK*^*shUSP10-2*^ organoids revealed that *Notum*, along with genes associated with a super competitor signature, such as *Dkk2*, *Dkk3* or *Wif1* [28, 29], were downregulated upon loss of USP10 (Supplementary Fig. S7b). This is in line with the observation that NOTUM was downregulated in patient derived CRC tumour organoids upon silencing of USP10 (Fig. 6f).

To further investigate in vivo if reduced WNT signalling, along with decreased NOTUM expression does affect tumour growth, we transplanted APK9 organoids stably expressing shNTC or shUSP10, respectively, in immune-competent C57Bl6/J mice (Fig. 7c). 24 weeks post-transplant mice were sacrificed, and tissue samples analysed. Mice transplanted with non-targeting control shRNA developed a large tumour, in line with previous reports for this genotype [39–41]. Loss of USP10, while resulting in tumour organoid engraftment, led to smaller lesions (Fig. 7d–f). Overall, tumour cells derived from shNTC organoids showed an enriched abundance of USP10 and β-Catenin, which is in line with our previous observations (Fig. 7e, f). Loss of USP10 led to a reduction in β-Catenin abundance (Fig. 7e, f). Remarkably, the NTC infected organoids showed morphologic resemblance to invasive tumours, while APK9^shUSP10^ showed an enriched abundance of mucus-secreting cells (Fig. 7e, star).

USP10 is required to maintain active WNT signalling, driving a stemness-like signature and is required to stabilise via β-Catenin and drive oncogenesis. Its loss ameliorates tumour growth abrogate the super competitor phenotype of APC and initiates the differentiation of tumours. Overall, USP10 presents a vulnerability for CRC, at point of induction and propagation.

## Discussion

Colorectal cancer is mostly driven by deregulated WNT signalling, resulting in hyper-activation of the proto-oncoprotein β-Catenin and constant transcription of its target genes [11]. This is predominantly initiated via loss of function mutations within the tumour suppressor gene *APC*; around 80% of all CRC patients harbour these mutations [4, 26]. It is noteworthy that truncation mutations vary regarding localisation and the therefore resulting remaining domains within the APC protein [7]. Recent studies have reported that tumour progression, loss-of-heterozygosity, and deregulation of WNT signalling are indeed affected by the length of the remaining tumour suppressor [42]. Furthermore, aberrant WNT signalling, caused by *APC* truncation, is a prerequisite for the establishing of a super competitor cell phenotype, manifesting CRC onset [28, 29, 33]. Despite our growing understanding of the underlying genetic causalities of colorectal cancer, the identification of mechanisms at the basis of this phenomenon and identification of suitable therapeutic targets presents a challenge that yet needs to be overcome [43].

One promising strategy is targeting enhanced onco-protein stability in cancer, in particular via the Ubiquitin Proteasome System (UPS) [44]. Despite the loss of either APC or mutations within the degron motive of β-Catenin, the WNT effector is still ubiquitylated, at least, in CRC-derived cell lines [7, 9]. Several E3 ligases have been reported in the past to ubiquitylate and thereby regulate the stability and activity of this proto-oncogene [18–22]. This opens an intriguing possibility to target the protein levels of β-Catenin by interfering with enzymes conferring stabilisation. As a potential therapeutically relevant druggable family of proteins DUBs are of interest, as this class of enzymes opposes substrate ubiquitylation and can contribute to protein stabilisation and activation [45].

In this study, we investigated the possibility to control the abundance of β-Catenin protein via DUBs that are relevant to CRC that is driven by loss of the tumour suppressor APC. Via unbiassed screen we discovered that USP10 enhances β-Catenin abundance. USP10 was reported to control the protein stability of TP53 [46, 47] and contribute to control of autophagy [48, 49], DNA damage [50, 51] and metabolic signalling [51, 52], all processes that tumour cells highly rely on [53]. Analysing publicly available patient data and samples from local CRC patients, we found that USP10 is frequently upregulated in human CRC tumours and is often co-expressed with β-Catenin. Intriguingly, by utilising a limited panel of human CRC cell lines, we observed that USP10 only interacted with β-Catenin when the truncation mutation within APC resulted in loss of the AAR domains. This observation was further corroborated by using CRISPR targeting of *APC* in otherwise *APC* non-mutant CRC lines. Microarray-based binding assays identified residues 7–21 of the unstructured N-terminus of USP10 to directly interact with β-Catenin. Comparison of the resulting structural model with the APC-β-Catenin complex lends a molecular explanation for the observed direct competition with APC. An overlapping binding site of APC and USP10 on β-Catenin would also rationalize the need for APC truncation for successful USP10 co-immunoprecipitation. Additionally, this observation could explain how APC mutations indirectly control β-Catenin abundance without affecting the ubiquitylation level per se. Taken together, our data extends the role of WNT signalling and β-Catenin in CRC and proposes patient stratification towards USP10 dependency. This is a novel path to the possibility of β-Catenin and allows to transform APC from a diagnostic marker towards an actionable vulnerability within CRC. Furthermore, our observation reports a direct interaction between USP10 and β-Catenin, confirming that both proteins do regulate tumour-intrinsic processes. Recently, this axis has been linked to drive NSCLC progression and metastasis [54]. Here, in contrast to our work, USP10 stabilized HDAC7, which in turn controlled β-Catenin abundance.

Recently, an alternative mechanism of regulation of the WNT pathway by USP10 was reported [55]. Here, during zebrafish development, USP10 contribute to the degradation of β-Catenin by recruiting and stabilising AXIN1 binding to β-Catenin. This is an interesting observation and highlights the possibility of substrate and functional specificity in a tissue specific context. This has been reported for additional enzymes belonging to the UPS family, such as USP28 [56–58]. In cancer, the function of USP10 as a tumour suppressor or proto-oncogene, is still not fully solved. Recent work demonstrated both functions in the same tumour entity, such as NSCLC [54, 59]. The reason for these differences is not well understood but its investigation and vigorous testing is required to elucidate the therapeutic potential of this deubiquitylase, USP10.

While USP10 regulates the abundance of β-Catenin by affecting its ubiquitylation, its loss had widespread consequences for *APC* truncated CRC. Altering USP10 abundance affected the expression of genes associated with proliferation, stemness and disease progression. USP10 imprints the cellular identity of cancer cells, as loss of USP10 altered the transcriptional profiles towards a non-transformed, differentiated state. Not only does USP10 control tumour intrinsic pathways and biological processes regulated by WNT, loss of USP10 suppressed cell death of non-transformed cells exposed to cultured medium from tumour organoids. Given that the super competitor signalling cascade [28, 29, 38] leads to the secretion of signalling molecules initiating cell death, loss of USP10 controls extrinsic signalling mechanisms and thereby opposes tumour growth. It is worth noting that the catalytic activity of USP10 was required for the effects observed; overexpression of the catalytic inactive form had no effect on global β-Catenin abundance nor on proliferation. Hence, the catalytic active site presents a suitable target site [60].

The observed effects in gene expression in the USP10 shRNA cell lines and organoids were a direct consequence of reduced β-Catenin protein abundance. These processes are highly conserved, as by employing *D.melanogaster* intestinal hyperproliferation models and silencing of *USP10* in the intestinal stem cell niche demonstrated that targeting USP10 in vivo, indeed, did ameliorate the *APC* phenotype caused by homozygous loss of the tumour suppressor (*APC*^*Q8/Q8*^) [35]. This observation is further supported by the organoid transplant model presented. The observed phenotype is in line with previous reports that inhibition NOTUM in established or transplanted CRC tumours does suppress tumour progression [40]. Here, pharmacologic targeting of USP10 by small molecule inhibitors [61] could extend the spectrum of possible intervention and open new avenues for CRC therapy.

Utilizing human primary patient material, colorectal cancer cell lines as well as genetically tailored murine organoids and *Drosophila melanogaster* as a model system for aberrant WNT signalling in the intestine, we unravelled a novel protein-protein interaction to which CRC are addicted. Taken together, our study provided robust in vitro and in vivo evidence that USP10 functions as a driver of CRC and could serve as a therapeutic relevant target in a distinct subset of *APC*-truncated patient cohort.

### Ethics approval and consent to participate

In vivo experiments concerning CRISPR mediated oncogenesis or the *Apc*^*min/+*^ mouse model were approved by the Regierung Unterfranken and the ethics committee under the license numbers 2532-2-555, 2532-2-556, 2532-2-694 and 2532-2-1002. The mouse strains used for this publication are listed. All animals are housed in standard cages in pathogen‐free facilities on a 12‐h light/dark cycle with *ad libitum* access to food and water. FELASA2014 guidelines were followed for animal maintenance.

Mouse experiments concerning AOM/DSS induced oncogenesis were reviewed and approved by the Regierungspräsidium Darmstadt, Darmstadt, Germany.

Human colorectal cancer samples, irrespective of sex, were obtained from the Pathology Department at the University Hospital Würzburg (Germany). Informed consent was obtained from all patients. Experiments agreed with the principles set out in the WMA Declaration of Helsinki and the Department of Health and Human Services Belmont Report. Samples were approved under Ethics Approval 17/01/2006 (University Hospital Würzburg).

P6T human colorectal cancer organoids were established in a previous study [31] and obtained a following material transfer agreement with Hubrecht Organoid Technology. The collection of colorectal tissue for the generation of CRC organoids was performed according to the guidelines of the European Network of Research Ethics Committees (EUREC) following European, national and local law. In all cases, patients signed informed consent after ethical committees approved the study protocols.

## Supplementary information

Supplementary Figures and Legends

Supplementary M&M
