# Correction: USP10 drives cancer stemness and enables super-competitor signalling in colorectal cancer

**DOI:** 10.1038/s41388-024-03262-3

**Published:** 2025-01-22

**Authors:** Michaela Reissland, Oliver Hartmann, Saskia Tauch, Jeroen M. Bugter, Cristian Prieto-Garcia, Clemens Schulte, Sinah Loebbert, Daniel Solvie, Eliya Bitman-Lotan, Ashwin Narain, Anne-Claire Jacomin, Christina Schuelein-Voelk, Carmina T. Fuss, Nikolett Pahor, Carsten Ade, Viktoria Buck, Michael Potente, Vivian Li, Gerti Beliu, Armin Wiegering, Tom Grossmann, Martin Eilers, Elmar Wolf, Hans Maric, Mathias Rosenfeldt, Madelon M. Maurice, Ivan Dikic, Peter Gallant, Amir Orian, Markus E. Diefenbacher

**Affiliations:** 1 Protein Stability and Cancer Group, University of Wuerzburg, Department of Biochemistry and Molecular Biology, Wuerzburg, Germany; 2 Mildred-Scheel Early Career Cancer Center, Wuerzburg, Germany; 3 Division of Signal Transduction and Growth Control, DKFZ/ZMBH Alliance, German Cancer Research Center (DKFZ), Heidelberg, Germany; 4 Oncode Institute and Department of Cell Biology, Center for Molecular Medicine, University Medical Center Utrecht, Utrecht, Netherlands; 5 Molecular Signalling Group, Institute of Biochemistry II, Goethe University Frankfurt, Frankfurt am Main, Germany; 6 Rudolf-Virchow-Center for Integrative and Translational Imaging, University of Wuerzburg, Wuerzburg, Germany; 7 Department of Biochemistry and Molecular Biology, University of Wuerzburg, Wuerzburg, Germany; 8 Faculty of Medicine, TICC, Technion Haifa, Haifa, Israel; 9 Cancer Systems Biology Group, University of Wuerzburg, Am Hubland, Wuerzburg, Germany; 10 Department of Internal Medicine I, Division of Endocrinology and Diabetes, University Hospital, University of Wuerzburg, Wuerzburg, Germany; 11 Institute for Pathology, University of Würzburg, Wuerzburg, Germany; 12 Angiogenesis & Metabolism Laboratory, Berlin Institute of Health at Charité, Universitätsmedizin Berlin, Berlin Germany and Max Delbrück Center for Molecular Medicine in the Helmholtz Association, Berlin, Germany; 13 Stem Cell and Cancer Biology Laboratory, The Francis Crick Institute, London, UK; 14 Department of General, Visceral, Transplantation, Vascular and Paediatric Surgery, University Hospital, Julius-Maximilians-University of Wuerzburg, Wuerzburg, Germany; 15 Amsterdam Institute of Molecular and Life Sciences, Amsterdam, Netherlands; 16 Institute of Lung Health and Immunity, Helmholtz Center, Munich, Germany; 17 German Center for Lung Research, DZL, Germany; 18 Ludwig Maximilian University Munich, Munich, Germany; 19 Present Address: Max Planck Institute for Molecular Genetics, Berlin, Germany

**Keywords:** Cancer genetics, Ubiquitylation

Correction to: *Oncogene* 10.1038/s41388-024-03141-x

The affiliation of the author Gerti Beliu has been corrected from “Institute of Lung Health and Immunity, Helmholtz Center, Munich, Germany” to “Rudolf-Virchow-Center for Integrative and Translational Imaging, University of Wuerzburg, Wuerzburg, Germany.” The original article has been corrected.

