# Supplementary material for: USP10 drives cancer stemness and enables super-competitor signalling in colorectal cancer
Source: Oncogene. 2024 Oct 23;43(50):3645–59. doi: 10.1038/s41388-024-03141-x (PMC11611742; doi:10.1038/s41388-024-03141-x)
Supplement: Supplementary file 1 — Supplementary Figures and Legends [file 41388_2024_3141_MOESM1_ESM.pdf]

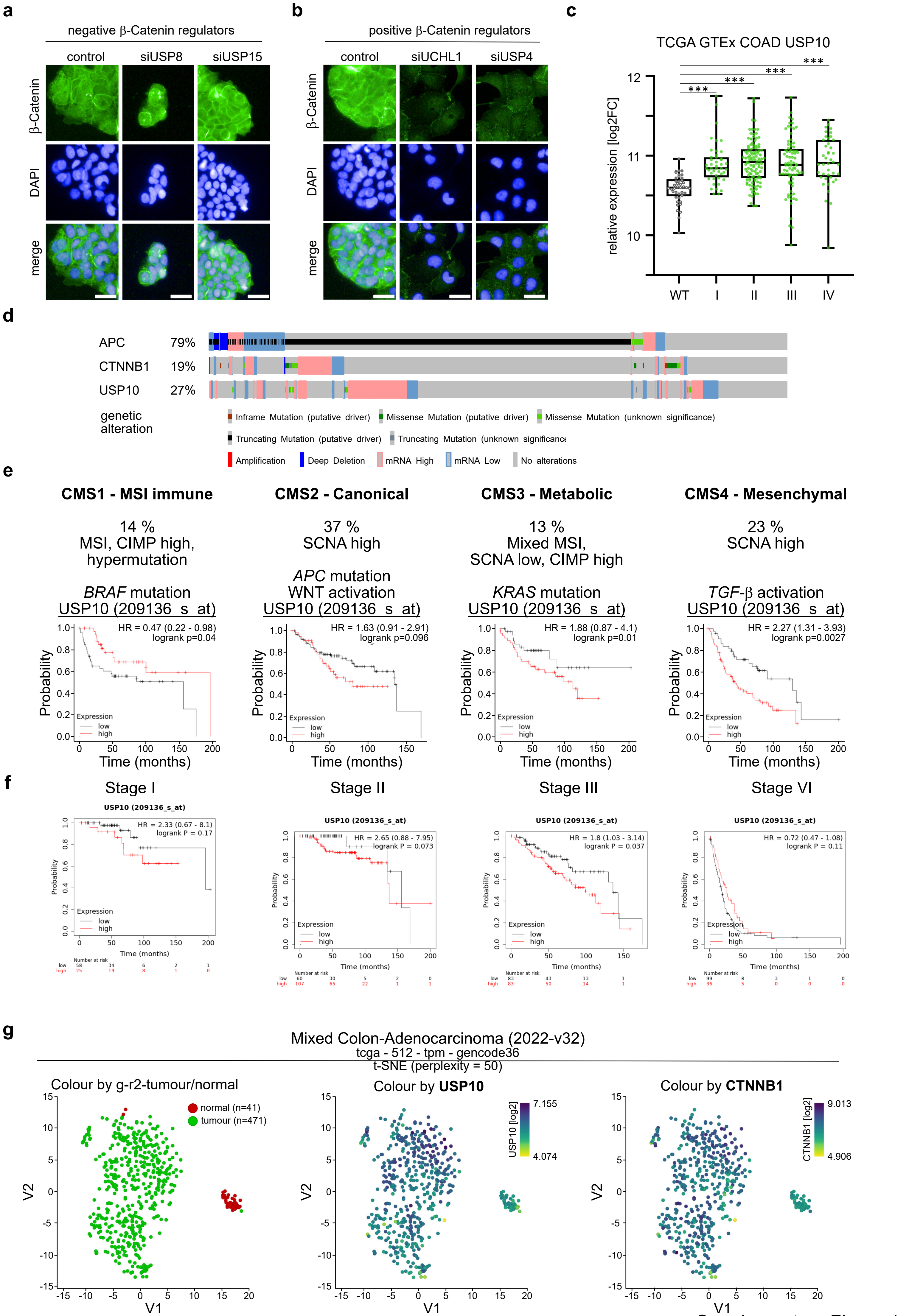

Supplementary Figure 1

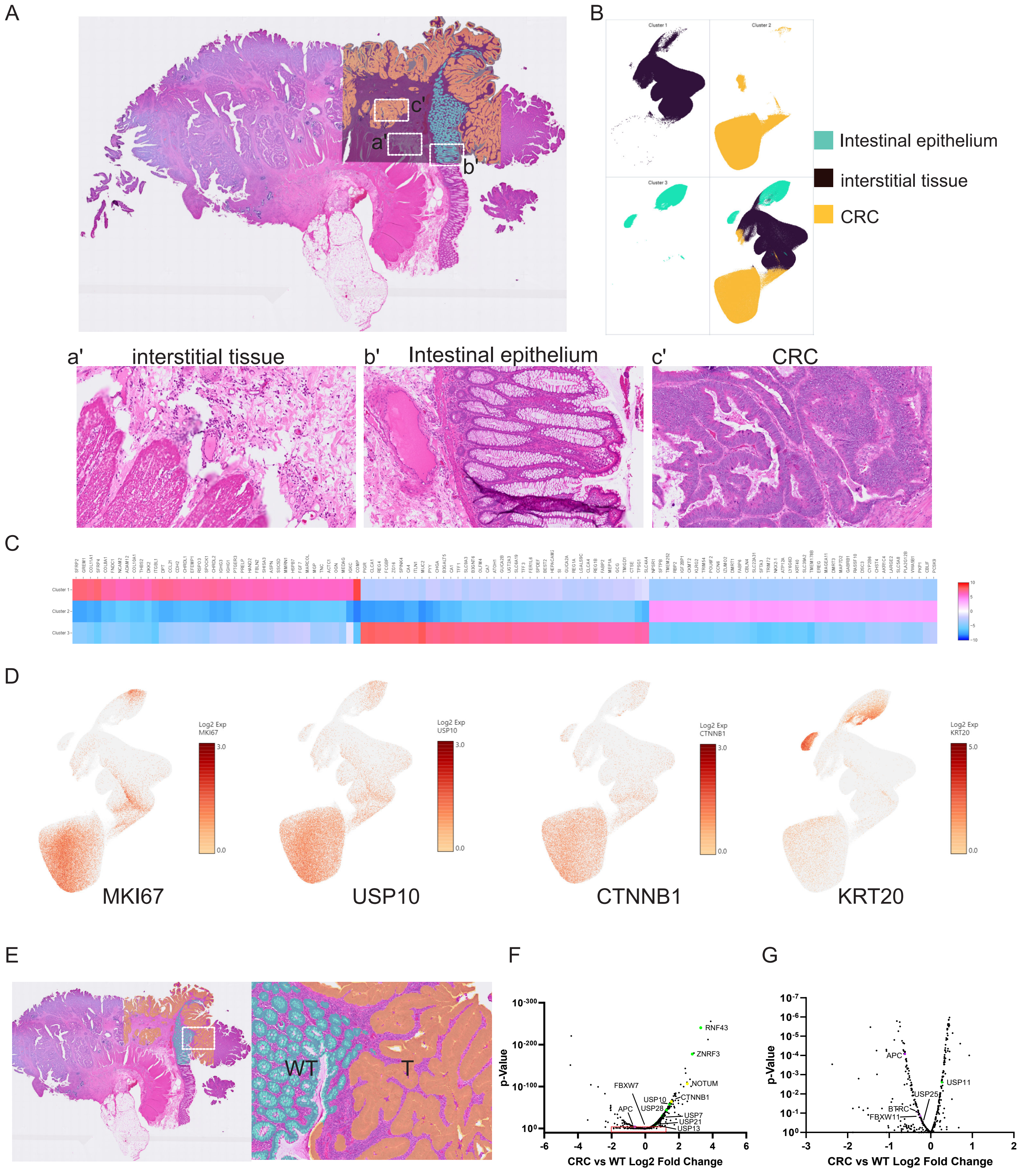

A
B
Intestinal epithelium
interstitial tissue
CRC
a' interstitial tissue
b' Intestinal epithelium
c' CRC
C
D
MKI67
USP10
CTNNB1
KRT20
E
WT
T
F
CRC vs WT Log2 Fold Change
p-Value
RNF43
ZNRF3
NOTUM
CTNNB1
FBXW7
USP10
USP28
APC
USP7
USP21
USP13
G
CRC vs WT Log2 Fold Change
p-Value
APC
USP11
USP25
BTRC
FBXW11

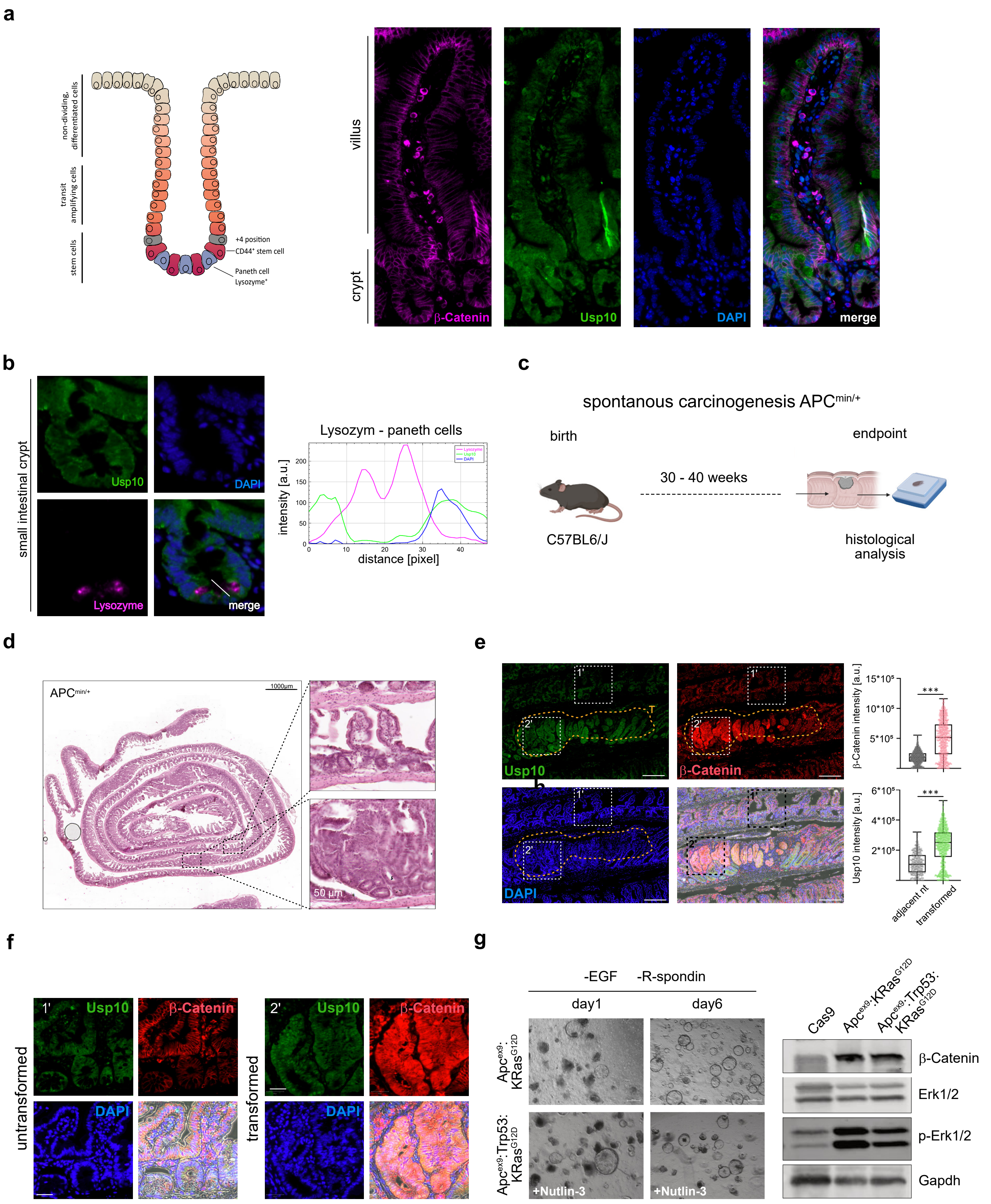

Supplementary Figure 2

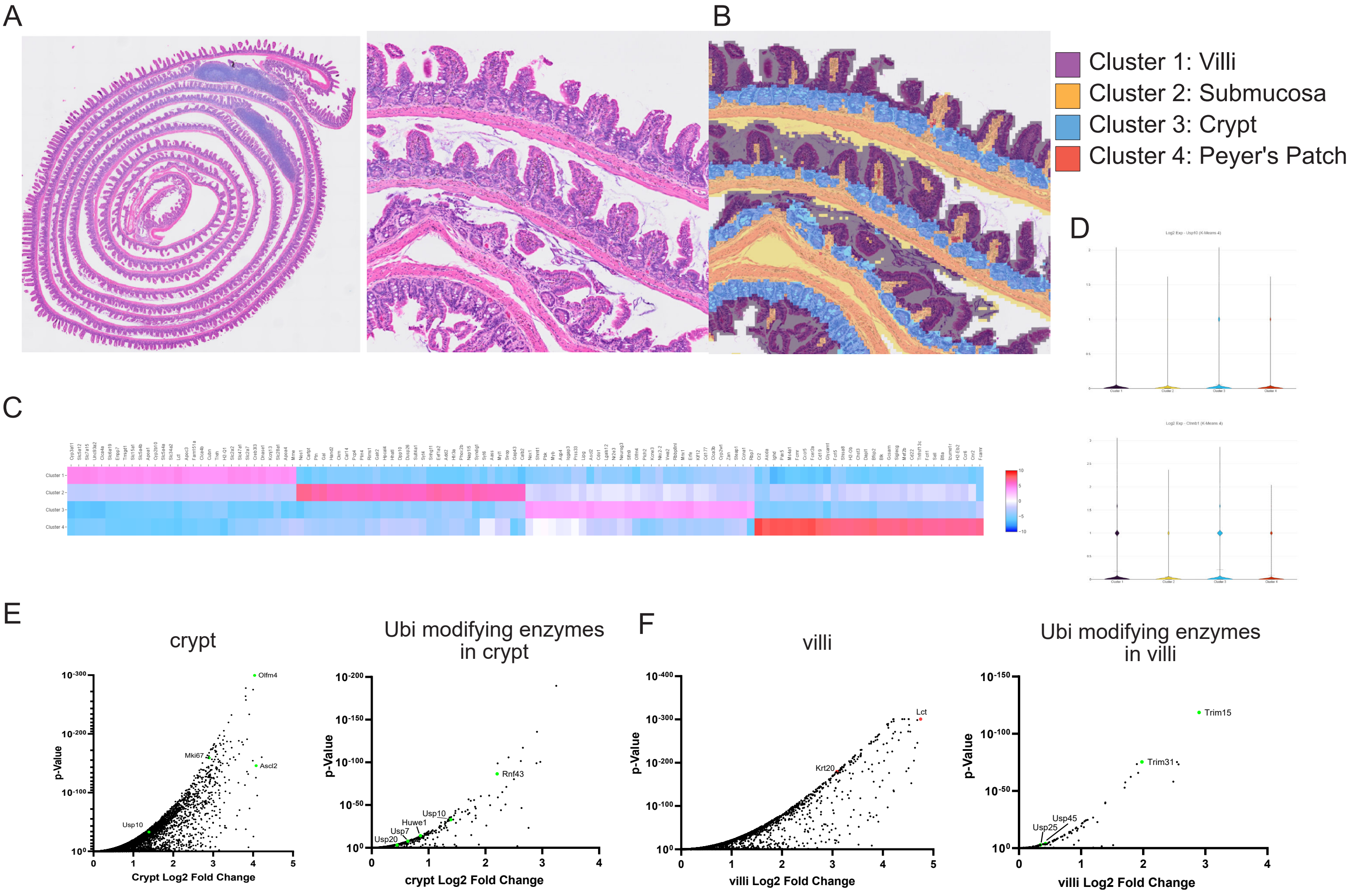
A
B
Cluster 1: Villi
Cluster 2: Submucosa
Cluster 3: Crypt
Cluster 4: Peyer's Patch
C
D
E
crypt
Olfm4
Mki67
Ascl2
Usp10
p-Value
Crypt Log2 Fold Change
Ubi modifying enzymes in crypt
Rnf43
Usp10
Huwe1
Usp7
Usp20
p-Value
crypt Log2 Fold Change
F
villi
Lct
Krt20
p-Value
villi Log2 Fold Change
Ubi modifying enzymes in villi
Trim15
Trim31
Usp45
Usp25
p-Value
villi Log2 Fold Change

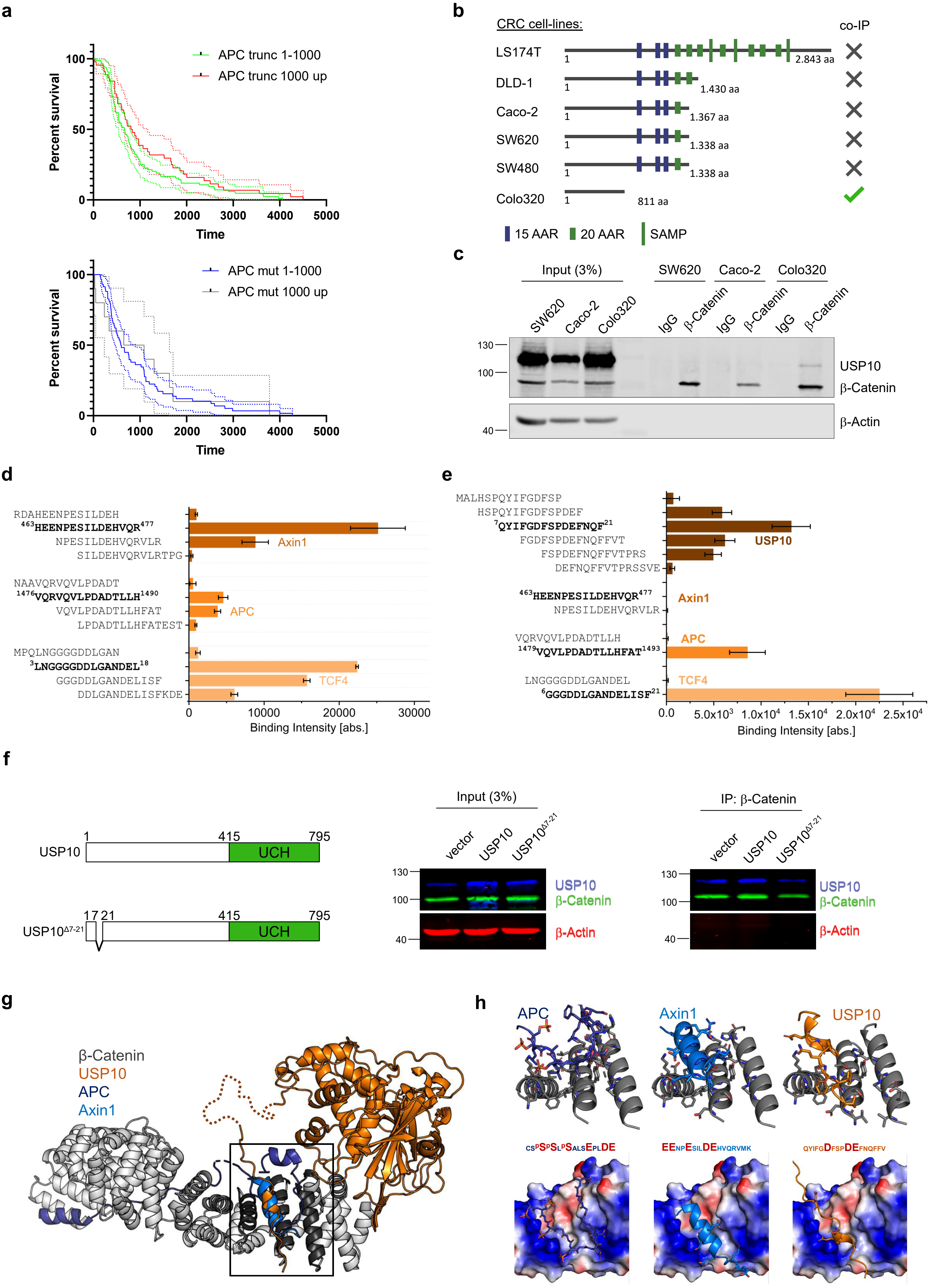

Supplementary Figure 3

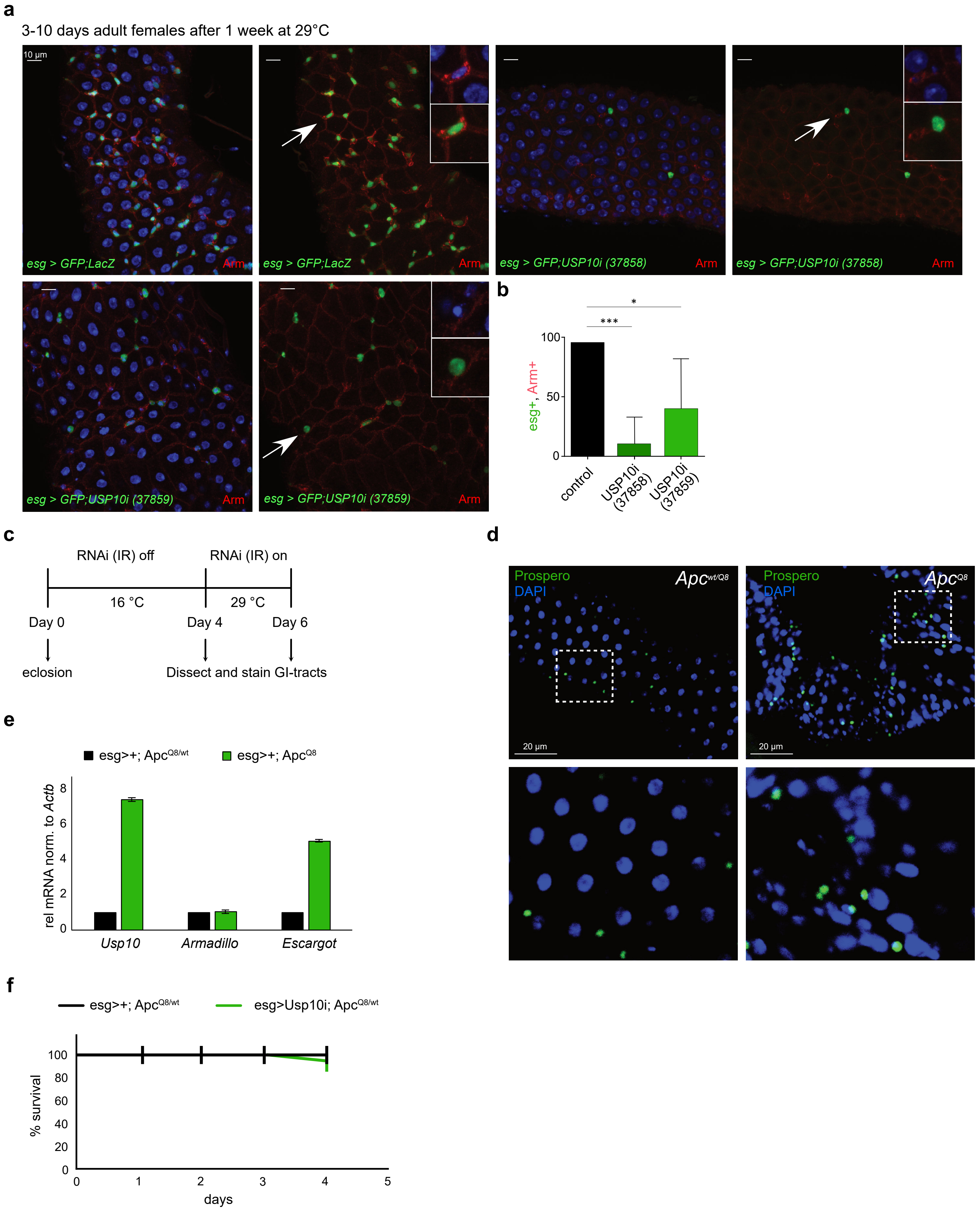

Supplementary Figure 4

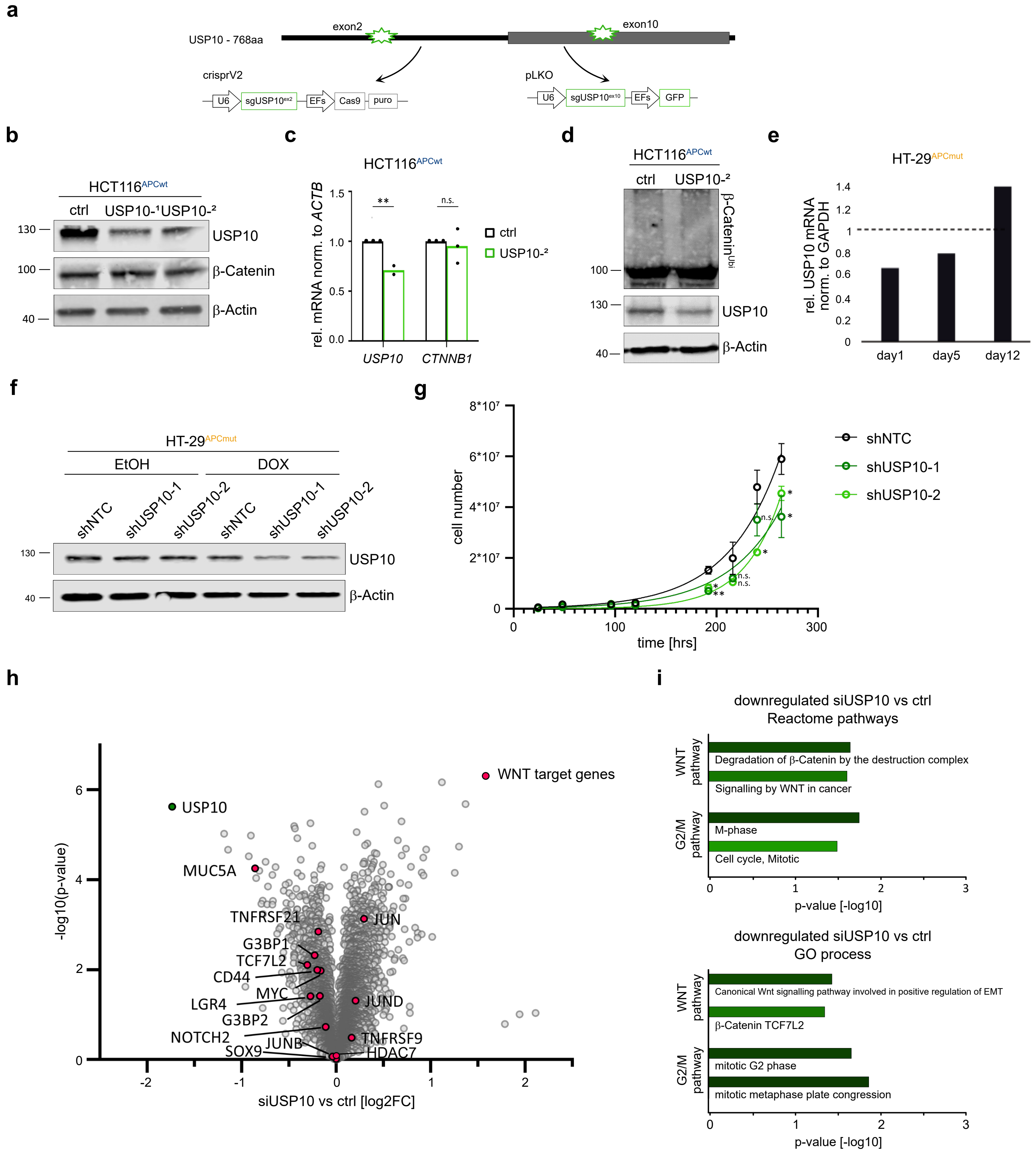

Supplementary Figure 5

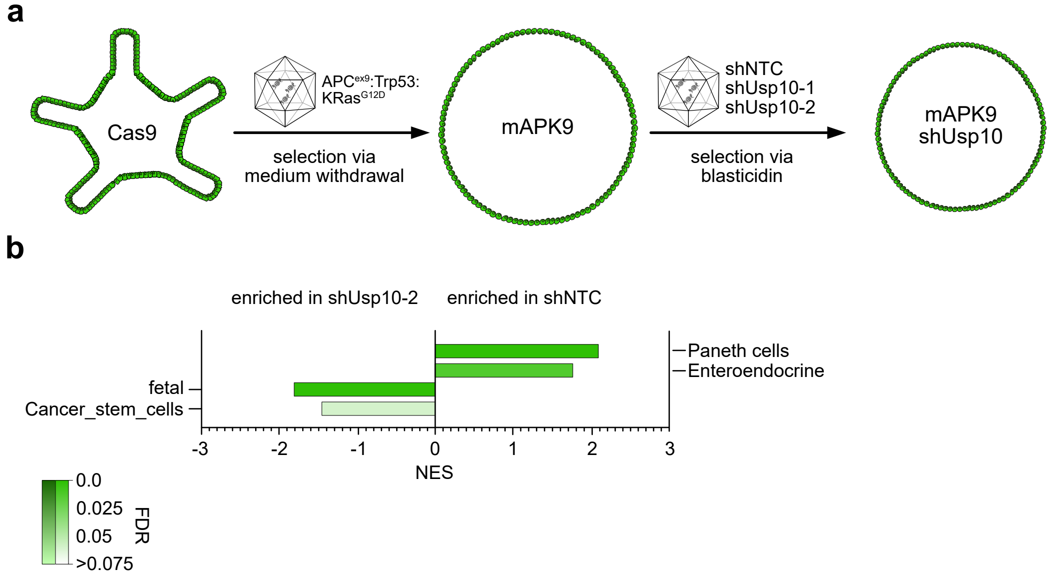
a
Cas9
APCex9:Trp53:
KRasG12D
selection via
medium withdrawal
mAPK9
shNTC
shUsp10-1
shUsp10-2
selection via
blasticidin
mAPK9
shUsp10
b
enriched in shUsp10-2
enriched in shNTC
Paneth cells
Enteroendocrine
fetal
Cancer_stem_cells
-3
-2
-1
0
1
2
3
NES
0.0
0.025
0.05
>0.075
FDR

a

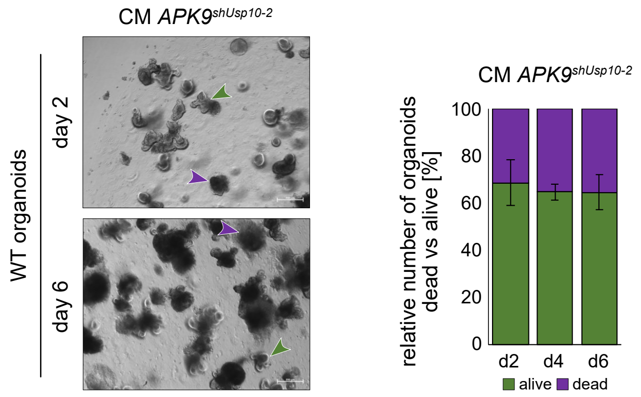
CM *APK9*$^{shUsp10-2}$
WT organoids
day 2
day 6
CM *APK9*$^{shUsp10-2}$
relative number of organoids dead vs alive [%]
100
80
60
40
20
0
d2
d4
d6
alive
dead

b

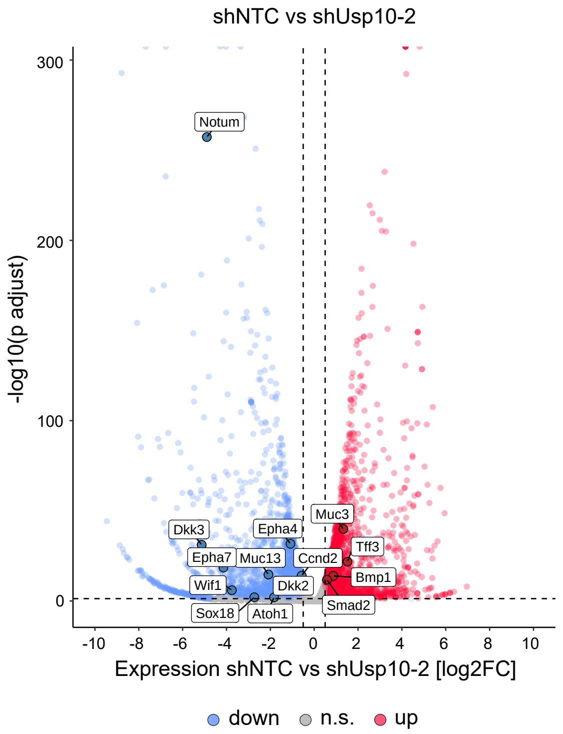
shNTC vs shUsp10-2
-log10(p adjust)
300
200
100
0
Notum
Dkk3
Epha4
Muc3
Epha7
Muc13
Ccnd2
Tff3
Wif1
Dkk2
Bmp1
Sox18
Atoh1
Smad2
-10
-8
-6
-4
-2
0
2
4
6
8
10
Expression shNTC vs shUsp10-2 [log2FC]
down
n.s.
up

Figure S7

Supplementary Figure legends:

Figure S1

a. Representative immunofluorescent images of endogenous β-Catenin (green) upon siRNA mediated knock-down of NTC (control) and the known negative regulators *USP8* and *USP15*. DAPI served as nuclear marker (blue). Scale bar indicates 25µm.
b. Representative immunofluorescent images of endogenous β-Catenin (green) upon siRNA mediated knock-down of NTC (control) and the known positive regulators *UCHL1* and *USP4*. DAPI served as nuclear marker (blue).
c. Expression of *USP10* in non-transformed (WT) and stage I – IV CRC samples. Publicly available data were extracted from *UCSC Xena*: GTEx (n=41) and COAD (Stage I n = 44, II = 111, III = 81, IV = 41). P-values were calculated using Mann-Whitney test. *p<0.05; ***p<0.001
d. Overview and percental occurrence of *APC, CTNNB1 and USP10* genetic alterations in CRC patients. Plot was generated using TCGA PanCancer data set using cBioportal.
e. Molecular Subtypes of CRC in humans. Shown are Kaplan-Meier plots of CRC patients, stratified for molecular subtypes, relative to USP10 expression. Data was generated using www.kmplot.com.
f. Schematic representation of CRC staging with corresponding Kaplan-Meier plots for CMSII-IV, according to stage for USP10 expression. Data was generated using www.kmplot.com
g. Color-coded representation of the Mixed Colon Adenocarcinoma (2022-v32) data set using t-SNE. For each representation, we report the sample classification (normal vs. tumor or normal vs adenoma, respectively) of either USP10 or CTNNB1. Data generated by R2: Genomics Analysis and Visualization Platform, using the TCGA and Shi datasets.

Figure S1 extended

a. Overview image of human CRC FFPE sample used for spatial transcriptomic analysis (10xGenomics Visium HD). Transcript capture area is highlighted as square box (green). White boxes indicate representative areas for the individual clusters (K-means = 3) for further analysis (a', b' and c', respectively).
b. UMAP representation of the clusters (K-means = 3), illustrating the profiles of intestinal epithelial cells (42.075 counts (8%); green), interstitial tissue (including fibroblasts, muscle cells; 292899 counts (54%); black) or colorectal cancer cells (210876 counts (39%); orange).
c. Heatmap of differentially expressed genes in the K-means = 3 clusters.
d. Representative UMAP of *MKI67*, *USP10*, *CTNNB1* and *KRT20* of A). Heatmap indicates the log2 expression of the indicated genes.
e. to g. Overview image and Volcano plot depicting ubiquitin modifying enzymes, including E1, E2, E3 and deubiquitylases, differentially expressed in colorectal cancer cells (orange) versus adjacent, non-transformed intestinal crypt cells (green). g. shows differentially expressed genes within the red box in f. Notably,

wild type intestinal cells express APC, BTRC, FBXW11 or USP25, while USP10, USP28, USP7, USP21 or USP13 were upregulated in CRC.

Figure S2

a. Schematical model of a WT small intestinal crypt with markers for paneth cells (Lysozyme) and stem cells (Cd44). Representative fluorescent images of WT intestinal crypt and villus. Endogenous Usp10 (green) and β-Catenin (magenta). DAPI served as nuclear marker (blue).
b. Representative immunofluorescent images of WT intestinal crypts of endogenous Usp10 (green) and crypt cell specific markers. Upper panel: Lysozyme (magenta) marks Paneth cells. Lower panel: Cd44 (magenta) labels stem cells. DAPI served as nuclear marker (blue). White line indicates stretch of fluorescence quantification. Histogram of fluorescence over indicated length.
c. Schematic representation of the spontaneous CRC model utilising C57BL6/J *Apc*$^{min/wt}$ mice. Loss of heterozygosity and CRC onset in the small intestine and colon appears withing 30-40 weeks post birth.
d. Haematoxylin and eosin (H&E) staining of intestines of *Apc*$^{min/wt}$ mice 30 weeks post birth. Insets highlight either non-transformed adjacent tissue or primary tumour.
e. Representative immunofluorescent images and quantification of intestines of *Apc*$^{min/wt}$ mice 30 weeks post birth, of endogenous Usp10 (green) and β-Catenin (red), respectively. DAPI served as nuclear marker (blue). Insets highlight either untranformed (1') or transformed (2') regions. P-values were calculated using Mann-Whitney U test. ***p <0.001.
f. Insets from Figure S1G. High magnification immunofluorescent images of intestines of genetically engineered APC$^{min/+}$ mice for Usp10 (green) and β-Catenin (red). DAPI served as nuclear marker (blue).
g. Representative brightfield images of infected organoids 1- and 6- days post infection. Successfully infected organoids were selected via withdrawal of distinct medium-components. ENR - EGF, Noggin, R-spondin. For *Trp53*$^{\Delta}$ selection, 10 µM of the MDM2 inhibitor Nutlin-3 was used for six days. Immunoblot analysis of infected AK and APK organoids compared to control. β-Catenin and p-ERK1/2 served as downstream targets for Apc truncation and KRas activation, respectively. Gapdh served as loading control.

Figure S2 extended

a. Overview image of murine FFPE sample used for spatial transcriptomic analysis (10x Genomics Visium HD). High magnification used to illustrate tissue composition.
b. K means = 4 clustering of murine intestinal tissue used to segment villi (violet), crypts (teal), submucosa (orange) or Peyer's patches (red, not shown in magnification).
c. Heatmap of differentially expressed genes in the K-means = 4 clusters.
d. Violin blots of Usp10 or Ctnnb1 expression in the K means = 4 clusters.
e. Volcano plots of upregulated genes in crypt cells relative to villi, or only depicting ubiquitin modifying enzymes (including E1, E2, E3 and deubiquitylases).

Highlighted are either stem cell markers (Olfm4, Ascl2, mKi67) or Usp10, along with Rnf43, Huwe1, Usp7 or Usp20, respectively (green dots).

f. Volcano plots of upregulated genes or only depicting ubiquitin modifying enzymes (including E1, E2, E3 and deubiquitylases), expressed in villi, relative to crypt cells. Highlighted are either villi markers (*Krt20, Lct*) or *Trim 15*, *Trim31* along with *Usp45* or *Usp25*, respectively (green dots).

Figure S3

a. Publicly available patient survival data of CRC patients were stratified according to APC truncation or mutation status. APC trunc 1-1000 n = 87 (green) and APC trunc 1000 up n = 42 (red); APC mut 1-1000 n=62 (blue) and APC mut 1000up n=8 (grey). Survival correlation analysis was performed using R2: Genomics Analysis and Visualization Platform, using the Tumour Colon - Smith dataset.
b. Schematic representation of truncating mutations reported in the *APC* gene in the CRC cell lines LS174T, DLD-1, Caco-2, SW480, SW620 and Colo320 and summary about observed co-IP of USP10 and β-Catenin. Dark blue box = 15 AAR domains, green small boxes = 20 AAR domains, large green boxes = SAMP domains. 15- and 20-AAR = β-Catenin amino acid repeats; SAMP = Axin binding sites.
c. Representative input and endogenous co-immunoprecipitation of USP10 and β-Catenin in human CRC cell lines SW620, Caco-2 and Colo320 (APCmut). IgG served as antibody specificity control. β-Actin served as loading control. Input represents 3% of total loading. n=1.
d. Overview of binding intensities of recombinant β-Catenin towards discreet regions of AXIN1, APC and TCF4. Colour code indicates binding intensity. Peptides with the globally highest binding intensity. Mean of n=3.
e. Same as d. but with the addition of peptides covering the putative binding site of USP10 on β-Catenin. Colour code indicates binding intensity. Peptides with the globally highest binding intensity. Mean of n=3.
f. Schematic representation of wild type USP10 and USP10$^{\Delta 7\text{-}21}$. Representative input and co-immunoprecipitation of β-Catenin and USP10 in human CRC cell line HT-29. β-Actin served as loading control. Input represents 3% of total loading.
g. Comparison of the AF2M predicted β-Catenin/complex and the crystal structure of β-Catenin bound to phosphorylated APC (PDB: 1TH1). USP10 (orange), β-Catenin (grey), AXIN1 (light blue) and APC (dark blue).
h. Highlighted is the common binding site on β-Catenin in which USP10, AXIN1 and APC are competing for binding.

Figure S4

a. Confocal images of adult females' midguts expressing UAS-GFP and the indicated transgenes under the control of the Escargot-Gal4 (Esg, GFP) promoter, that direct *GAL4* expression to progenitor cells. DAPI (blue) marks nuclei and arrows point to cells shown in insets. Elimination of USP10 in progenitor cells using the indicated UAS-RNAi lines, but not control, results in reduced number of progenitor cells and reduced progenitors expressing Armadillo (GFP+, Arm+, red).

b. Quantification of esg+ and Arm+ cells from a. Error bars represent standard deviation of n=3 independent experiments. Significance was calculated using students t-test. *p<0.05; ***p<0.001.
c. Schematic overview of the intestinal hyperplasia survival study using the heat shock induced siRNA expression in *D.melanogaster*.
d. Immunofluorescent images against endogenous Prospero (green) from midguts of *Apc*$^{Q8/+}$ or *Apc*$^{Q8/Q8}$ flies. Prospero was used to distinguish between big and polyploid EC-like cells, small Prospero- (ISC/EB) and small Prospero+ (EE) cells. DAPI served as nuclear marker (blue).
e. qRT-PCR analysis of the expression of d*Usp10*, *Armadillo* and *Escargot* in midguts isolated from either *Apc*$^{Q8/+}$ or *Apc*$^{Q8/Q8}$ flies. Rel. mRNA was normalised to *Actb*. Error bars represent standard deviation of n=3. Significance was calculated using unpaired t-test. *p-value<0.05; **p-value<0.005; ***p-value<0.001.
f. Kaplan Meier plot of adult *D.melanogaster* survival of the indicated genotypes. n=35.

Figure S5

a. Schematic model of *USP10* targeting strategy via CRISPR/Cas9. A double guide approach, targeting exon 2 and 10 of *USP10*, with GFP and puromycin for selection was utilised.
b. Representative immunoblot against endogenous USP10 and β-Catenin in APC wild-type HCT116 cells upon CRISPR mediated depletion of USP10. Two different cell pools (USP10^{-1}, USP10^{-2}) along with non-targeting control cells are shown. β-Actin served as loading control. n=2.
c. Quantitative RT-PCR of *USP10* and *CTNNB1* expression of HCT116 USP10^{-2}. Error bars represent standard deviation of n=3 independent experiments. Significance was calculated using students t-test. **p<0.005; n.s.=non-significant.
d. Tandem Ubiquitin Binding Entity (TUBE) assay of endogenous poly-ubiquitylated proteins, followed by immunoblotting against endogenous β-Catenin in HCT116 USP10^{-2}. β-Actin served as loading control. n=2.
e. Quantitative RT-PCR of *USP10* expression in HT-29 USP10$^{\Delta}$ cells over time.
f. Immunoblot for two different DOX-inducible shRNAs against *USP10* and control shRNA in HT-29 cells after 4 days of 0.5 µM DOX and EtOH control treatment. Endogenous USP10 and β-Catenin was blotted. β-Actin served as loading control.
g. Growth-curve of shUSP10-1 and -2 compared to shNTC HT-29 cells. Error bars represent standard deviation of n=3 independent experiments. Significance was calculated using multiple paired t-tests. *p<0.05; **p<0.005; n.s.=non-significant.
h. Volcano-plot of up- and downregulated proteins upon siRNA mediated knock-down of USP10 in HT-29 cells. Proteins related to WNT signalling are highlighted in pink.
i. PANTHER pathway analysis from (h).

Figure S6

a. Schematic representation of two-step Cas9 organoid infection strategy via AAVs. sgRNAs targeting *Apc* in exon 9, *Trp53* and *KRas* with corresponding HDR-template under the control of the U6-Promoter were used to transform Cas9

organoids. Successfully selected APK organoids were then infected with AAV carrying shUsp10-1, -2 and shNTC and selected via blasticidin.

b. Gene set enrichment analysis of intestinal specific gene sets, deregulated in shUsp10-2 compared to shNTC APK9 organoids.

Figure S7

a. Representative brightfield images of wild-type (WT) organoids cultured in *APK*$^{shUsp10\text{-}2}$ conditioned medium (CM), supplemented with EGF and R-Spondin, for up to 6 days. Purple arrows indicate dead organoids, green arrows indicate live organoids. Quantification: Dead and alive organoids were counted, and bar graphs represent percentage of alive vs dead organoids. Error bars represent standard deviation calculated from n=3 independent experiments.

b. Volcano-plot of differential expressed genes in shUsp10-2 compared to shNTC organoids. Up- and down-regulated genes are highlighted in red and blue, respectively. Genes-of-interest are labelled.
